# Fabrication and electrochemical study of copper doped zinc sulfide/graphene nanocomposites for supercapacitors†

**DOI:** 10.1039/d5ra01710f

**Published:** 2025-07-11

**Authors:** Shumaila Saleem, Sadia Khalid, Aalia Nazir, Yaqoob Khan, Imtiaz Ahmad, Rameesha

**Affiliations:** a Institute of Physics, The Islamia University of Bahawalpur Bahawalpur 63100 Pakistan; b Nanosciences & Technology Department, National Centre for Physics, Quaid-e-Azam University Campus Shahdra Valley Road Islamabad 45320 Pakistan sadiabzu@gmail.com sadia.khalid@ncp.edu.pk; c Department of Physics, University of Science and Technology (UET) Lahore 54890 Pakistan; d Department of Physics, Faculty of Science, University of Sialkot Sialkot 50700 Pakistan

## Abstract

Transition metal sulfides exhibit excellent electrochemical performance and electrochemical energy storage capacity. Herein, we present high-capacity supercapacitor electrode based on copper doped zinc sulfide/graphene (ZCG) synthesized by co-precipitation method. Various techniques have been employed to characterize the ZCG nanocomposite including electrochemical measurements. The ZCG nanocomposites exhibit high crystallinity and phase purity. In three-electrode system and 1 M aqueous KOH solution, the prepared ZCG electrodes are evaluated using galvanostatic charge–discharge cycles (GCD), cyclic voltammetry (CV), and electrochemical impedance spectroscopy (EIS). The ZCG electrode exhibits an ultrahigh specific capacitance of 2295 F g^−1^ at a relatively low scan rate of 5 mV s^−1^ from CV and 743 F g^−1^ at 100 A g^−1^ from GCD, with exceptional cycling stability (93% capacity retention after 1000 cycles). Furthermore, the ZCG10 symmetric coin cell exhibits a specific capacitance, energy density, power density of 130.8 F g^−1^, 18 W h kg^−1^, 2400 W kg^−1^ at current density of 1.2 A g^−1^ in a 1 M KOH solution from GCD. The ZCG hybrid electrode material can be predicted a potential hybrid electrode material for the future development of energy storage devices.

## Abstract 2

Transition metal sulfides offer high electrochemical activity, multiple redox sites, and superior energy storage capacity, making them ideal for next-generation supercapacitors with enhanced performance and stability.

## Graphical abstract

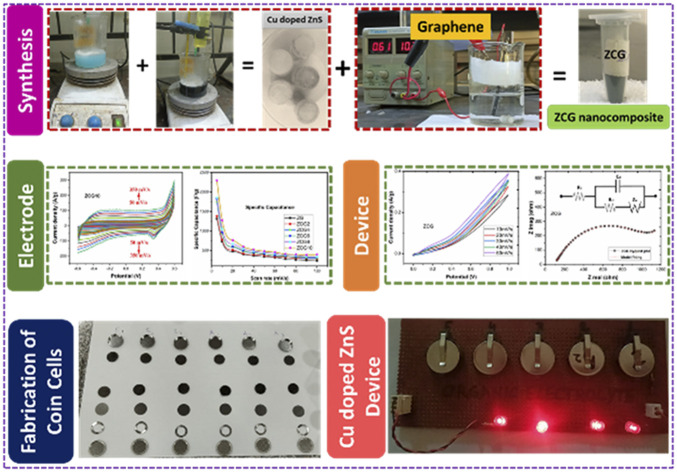

## Introduction

For decades, scientists have been working hard to develop electrochemical energy storage systems with ideal capacitance values (in the hundreds of F g^−1^). The initial investigations have shown the unexpected potential of supercapacitors and established a novel way for the advancement of electrochemical energy storage devices.^1^ Renewable energy technologies are considered as significant opportunities for the future and require further advancement in this century to assume a major role in energy production. Numerous energy storage technologies are now available, each with varying levels of development.^2^ Energy storage systems involve a range of energy storage technologies such as redox flow batteries, rechargeable batteries, fuel cells, and supercapacitors.^3,4^

Supercapacitors, also known as electrochemical capacitors, store electric charges through static adsorption (electric double-layer capacitance) or redox reaction (pseudocapacitance) mechanisms. Supercapacitors possess distinctive features including high power density, rapid charging or discharging rates, extended lifespan, and secure operation. Because of environmentally friendly electrode materials, supercapacitors are extensively employed in many commercial applications such as braking systems for automobiles and elevators, electrical grid stability, electric buses, computer memory backup, and consumer electronics like Samsung's stylus pen.^5,6^

Transition metal-based electrode materials are very favourable electrodes for supercapacitors (SCs) due to their remarkable electrochemical stability, specific power, and rapid charging/discharging rates.^7,8^ Transition metal sulfides (TMSs) are gaining recognition due to their unique electrical and electrochemical characteristics. Ongoing research has prioritized the use of readily available and non-harmful materials for electrode production, with the aim of achieving cost-effectiveness. TMSs have improved electrochemical performance compared to transition metal oxides (TMOs) due to the substitution of oxygen atoms with sulfur atoms. The characteristics and effectiveness of TMSs are strongly linked to their configurations, dimensions, and surface morphologies.^9,10^ However, these metal sulfides encounter challenges such as limited electrochemical stability, short lifespan, and low energy density.^11^

Graphene, an extremely thin sheet consisting of carbon atoms connected in a planar arrangement with sp^2^ bonds, has garnered significant interest because of its exceptional electrical, mechanical, thermal, and optical characteristics. The integration of graphene with inorganic materials including metals, metal oxides, and metal sulfides has garnered significant attention in recent years due to their versatile capabilities. Various synthesis methods have been developed and implemented to achieve graphene-based nanocomposites with controlled features.^12^ It has acquired significant attention because to its non-toxicity, cost-effectiveness, environmental friendliness, and absence of heavy metals.^6,12,13^ Graphene exhibits a significant ability to transport charge and is compatible with living organisms, enabling effective separation of charges in composite structures.^14^ Graphene, due to its extensive surface area, can serve as a conductive for the separation and transmission of electrons and holes.

ZnS possesses a wide band gap (3.5–3.8 eV), making it a suitable host for metal doping, including substitutional doping at the zinc sites. Recent reports indicate that the cubic form of ZnS is the most stable crystallographic form at room temperature. However, it undergoes a transformation to the hexagonal form when subjected to heat treatment at temperatures over 1020 °C.^15^ Moreover, doping with transition metals can significantly enhance electrochemical performance in binary metal sulfides, resulting the superior electrochemical performance compared to single transition metal sulfides. Therefore, it is more important to enhance the electrochemical efficiency of pseudocapacitors by the enhancement of their electrode internal structure, morphology and surface area.^16,17^

In recent years, there has been significant interest in weakly magnetic and paramagnetic ion-doped semiconductors.^18^ Doping improves semiconductor properties by giving a better way for controlling their optical, electrical, and ion transport properties.^19,20^ Cu^2+^ is a favourable metallic option for introducing impurities into zinc metal, beside chalcogenide. This is due to the almost same ionic radii of both cations, which is a crucial feature in the doping process.^21–24^ By adding the small amount of Cu into ZnS lattice, both the elemental composition and morphology can be modified. Furthermore, the material attains exceptionally high electrochemical performance, which offers a promising direction for development of future high performance electrode materials.^25^ Asfaram *et al.*^26^ studied the synthesis and analysis of Cu^2+^ doped ZnS on activated carbon for the purpose of simultaneous ternary adsorption of dyes. Hussain *et al.*^16^ studied the improved electrochemical performance and cycle stability of Cu doped ZnS nanostructures, which was due to interconnecting polyhedron-like structures, and binder-free electrode design.

Electrochemical performance of a supercapacitor electrode has stayed a challenge. Aim of this work is to improve electrochemical performance of electrode material by rational designing of electrode material. This can be achieved using strategies like doping and introduction of carbon nanomaterial. The combination of Cu doped ZnS and graphene creates a synergistic effect with enhanced electrochemical properties. Graphene, known for its high surface area, electrical conductivity, and mechanical strength, can improve the overall performance of the supercapacitor electrode when combined with Cu doped ZnS. It has been observed that a high specific capacitance and longer cyclic stability can be attained using graphene.^27,28^

The other challenge is agglomeration of metal sulfide nanoparticles that lowers the specific capacitance of the electrode material by reducing active surface area.^29^ In present work, Triton X-100 (TX-100), a non-ionic surfactant has been used that played an indispensable role in controlling the particle size of the synthesized ZnS nanoparticles, acting as a surfactant that limited agglomeration and promoted uniform nucleation.^30^ The presence of TX-100 during the coprecipitation process led to smaller and more uniform ZnS particles, likely due to its ability to stabilize the growing crystals and prevent uncontrolled aggregation.^31^

In this work, pure ZnS, copper-doped ZnS (ZCS) nanoparticles, and copper-doped ZnS/graphene (ZCG) are fabricated *via* the simple and economic coprecipitation method. In this study, the optimal Cu doping concentration in ZnS has been evaluated in order to improve its properties in energy storage applications such as supercapacitor. In the nanoscale regime, graphene assisted in the manipulation of electronic energy states and surface area. Due to these qualities, ZCS and ZCG are viable materials for energy applications. To validate the formation of ZCS and ZCG nanoparticles, structural, morphological, optical, and thermal properties, specific surface area and chemical state of samples have been conducted, using powder XRD, FE-SEM, DRS, FTIR, TGA, BET and XPS. Using CV, GCD and EIS, electrochemical behaviour has been investigated. Furthermore, effect of doping concentration on the electrochemical performance of nanocomposites has been comprehensively studied. The overall findings revealed improved electrochemical performance of composites as compared to pure ZnS. This valuable insights about the ZCG nanocomposite opens the economic avenues towards facile synthesis of highly efficient reproducible supercapacitor electrode materials.

## Experiment

### Material and methods

#### Materials

The analytical reagents grade of zinc nitrate hexahydrate (Zn(NO_3_)_2_·6H_2_O), sodium sulfide (Na_2_S), copper nitrate trihydrate (Cu(NO_3_)_2_·3H_2_O) as a dopant, Triton X-100 (TX-100) was supplied from Sigma Aldrich. Graphite rod, ammonium sulfate ((NH_4_)_2_SO_4_) was used for graphene preparation. Carbon black, *N*-methyl-2-pyrrolidone (NMP), aluminum foil, polyvinyl alcohol (PVA), ethanol was used for electrode preparation.

#### Preparation of ZnS

ZnS were synthesized using the simple coprecipitation technique. The ZnS nanoparticles were synthesized as follows: Zn(NO_3_)_2_·6H_2_O was dissolved in 50 ml of deionized water under continuous stirring to prepare 1 M solution and then appropriate amount of TX-100 was added dropwise, and temperature was maintained at 80 °C. The Na_2_S was added to 50 ml of deionized water under continuous stirring to prepare 2 M solution. The Na_2_S solution was then poured dropwise into previously prepared solution. After the reaction was completed, off-white precipitates were formed. Then this mixture was centrifuged at 5000 rpm for 5 min, the pH was maintained neutral and material was dried at 80 °C for 12 h in oven.

#### Preparation of Cu doped ZnS (ZCS)

**Fig. 1:**
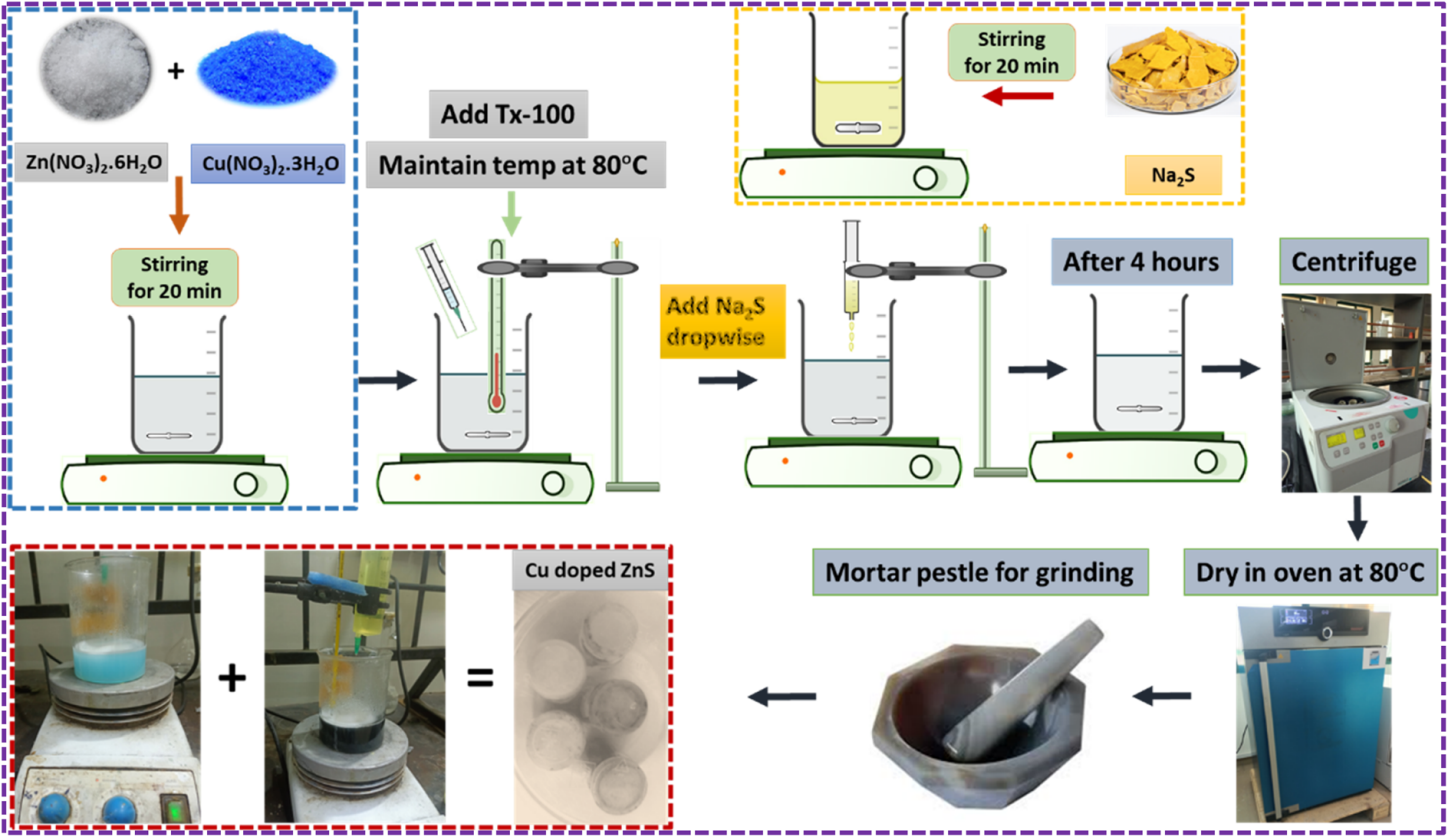
Schematic diagram of Cu doped ZnS (ZCS) nanomaterial by Coprecipitation Method.

Cu-doped ZnS nanoparticles were synthesized using the same coprecipitation procedure (Fig. 1). The following steps were performed during synthesis of Cu doped ZnS nanoparticles followed by 2, 4, 6, 8, and 10 mol% of Cu. The prepared Cu:ZnS powders were labelled as ZS pure (0 mol%), ZCS2 (2 mol%), ZCS4 (4 mol%), ZCS6 (6 mol%), ZCS8 (8 mol%), and ZCS10 (10 mol%).

#### Preparation of graphene

**Fig. 2:**
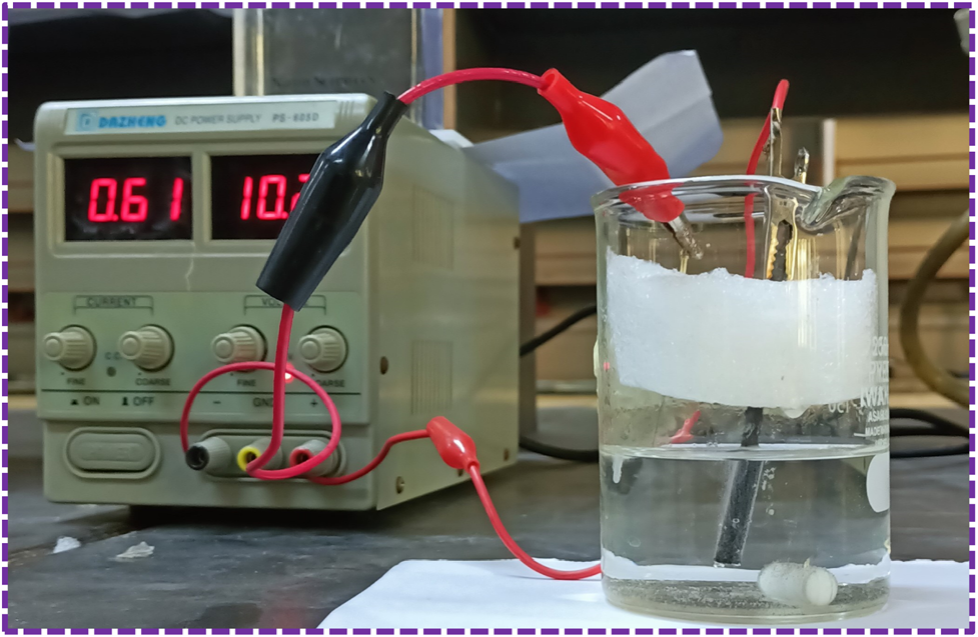
Graphene synthesis by electrochemical exfoliation.

The preparation of graphene *via* electrochemical exfoliation process has been described in ref. 32 (Fig. 2).

#### Preparation of Cu doped ZnS/graphene (ZCG)

**Fig. 3:**
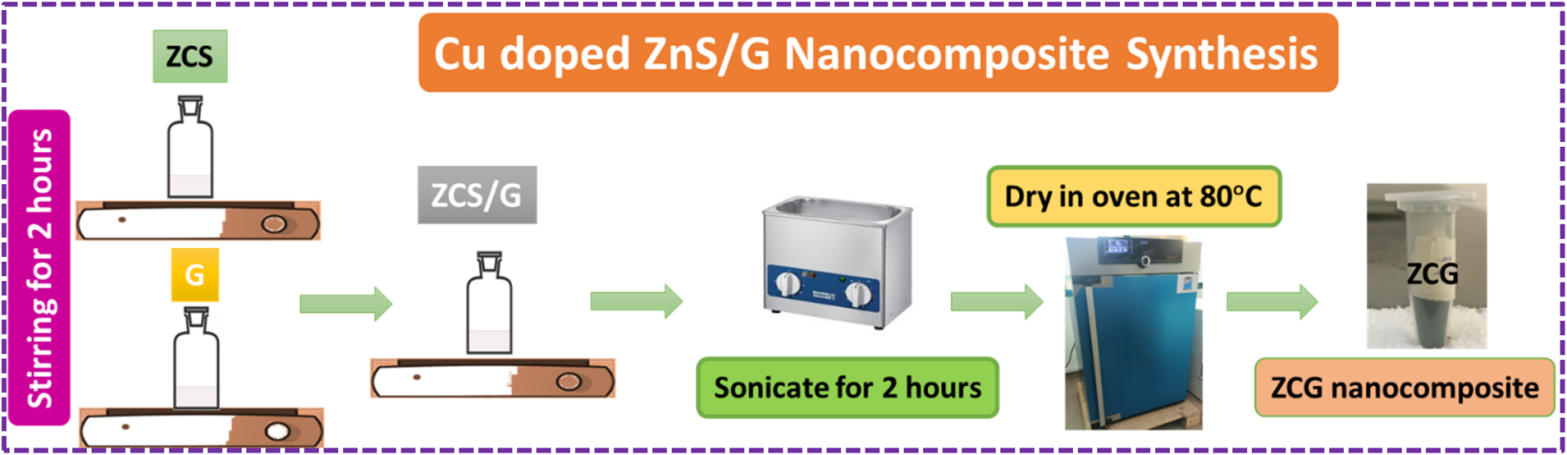
Schematic diagram of ZnS/G nanocomposite.

ZCG nanocomposite was prepared using 0.95 : 0.05 ratio of ZS or ZCS to G suspensions in ultrasonic bath (Fig. 3). The samples were labelled as ZG, ZCG2, ZCG4, ZCG6, ZCG8, ZCG10.

#### Electrode preparation

Glassy carbon electrode (GCE) was used to analyse the electrochemical performance of the active electrode materials instead of nickel foam (NF) substrate. This provided a realistic approach to evaluate the practical performance and electrochemical stability of the samples.^33^ The area of a GCE was carefully cleaned a mirror-like surface by using alumina slurry. After washing the GCE with deionized water, it was sonicated in a mixture of deionized water and ethanol (1 : 1) and then dried in the air. The fabrication of working electrode was done using a simple one-step sonication procedure. The suspension of ZCG nanocomposite, carbon black (conductive agent), and PVA as binder was prepared by using sonication method for 1 hour and deposited onto a GCE. The mass loading of working electrode was 0.5 μg. Working electrode of ZCG nanocomposite was dried and used for electrochemical measurements.

#### Device preparation

NF was selected as a substrate material for device fabrication as a current collector rather than aluminium foil. Although NF has limitations of oxygen and hydrogen evolution reactions, yet it offers high electrical conductivity, larger surface area, better durability, enhanced specific capacitance as well as high mass loading^34^ Performance of the device depends on the modification procedure of the NF with active material. The surface of a NF was carefully washed with water and ethanol mixture and then dipped into the diluted HCl for 30 s. After washing nickel foam in deionized water, it was sonicated for 5 min in mixture of deionized water and ethanol (1 : 1) and then dried in oven. For ZCG10 coin cell fabrication, active material (90%), carbon black (5%) and binder (5%) were used to fabricate electrodes.

### Characterizations

#### X-ray diffraction (XRD)

The crystallographic information was evaluated using XRD technique (Bruker D8 Advance) with a Cu-Kα radiation wavelength (*λ*) = 1.54060 Å over 2*θ* range between 15° and 70° with step size of 0.2°.

Bragg's equation was used to calculate interplanar spacing (*d*) between diffracting planes in pure ZnS, ZCS, and ZCG nanocomposites:

1 2*d* sin *θ* = *nλ*

where “*θ*” denotes scattering angle, “*d*” is interplanar spacing, “*n*” is a positive integer, and “*λ*” is wavelength of X-rays.

By using Scherrer eqn (2) the crystallite size was calculated.2
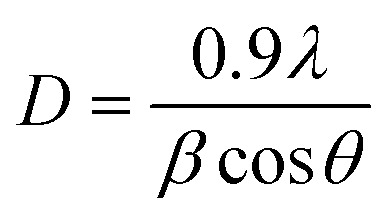
where “*β*” represents the full width half maximum (FWHM).^35^

#### Field emission scanning electron microscopy (FE-SEM) and energy dispersive X-ray (EDX)

The morphological study of ZnS, ZCS and ZCG nanocomposites were performed by ZEISS FESEM at 15 kV voltage with Oxford EDX for elemental distribution.

#### Diffuse reflectance spectroscopy (DRS)

Reflectance data of the as prepared samples was collected by PerkinElmer UV-vis spectrometer.

The band gap energy was evaluated from diffuse reflectance spectra.3
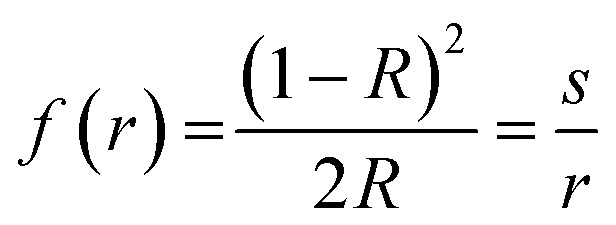
where “*R*” is the absolute reflectance of sample, “*s*” represents the absorption constant and “*r*” represents the scattering constant.^36^

The relationship between band gap and the absorption coefficient of a semiconductor material is as follows:

4 (*αhν*)^*n*^ = *A*(*hν* − *E*_g_)

where “*α*” is absorption coefficient, “*hν*” is photon energy, “*A*” is a constant and “*E*_g_” is the band gap, “*n*” varies according to the type of transition: *n* can take on the values 2, 1/2, 2/3, and 1/3, which correspond to the permitted indirect, permitted direct, prohibited direct, and prohibited indirect transitions.^37^

#### Fourier transform infrared spectroscopy (FT-IR)

FT-IR spectra in attenuated total reflectance (ATR) mode by Bruker TENSOR 27 were measured at wavenumber range (4000–600 cm^−1^) with resolution of 4 cm^−1^.

#### Thermogravimetric analysis (TGA) and differential scanning calorimetry (DSC)

TGA (weight loss%) and DSC (heat flow) were executed in nitrogen environment by METLLER Toledo TGA of ZCS10 and ZCG10 with sample weight 4.9244 mg and 3.7513 mg within the temperature range of 50–950 °C.

#### X-ray photoelectron spectroscopy (XPS)

A Scienta-Omicron system equipped with a micro-focused monochromatic Al K-Alpha X-ray source was utilized to get the XPS data. The data collection was performed using Matrix software, and analysis of the data was conducted with CasaXPS software, utilizing XPS fitting techniques. Utilizing Gaussian–Lorentzian line shape, we were able to fit detailed spectra, incorporating shiny background adjustments.

#### Brunauer–Emmett–Teller (BET) analysis

The BET analysis was employed to measure the surface area, pore size and pore volume of as-prepared samples at liquid nitrogen temperature, utilizing nitrogen adsorption–desorption isotherms on a Micromeritics ASAP 2020 apparatus.

#### Electrochemical measurements

CV, GCD, and EIS were performed on the synthesized materials using an electrochemical workstation Gamry reference 3000 potentiostat with a three-electrode setup in 1 M KOH electrolyte. ZG, ZCG2, ZCG4, ZCG6, ZCG8, ZCG10 were used as the working electrode, platinum wire as counter electrode, and Ag/AgCl was used as reference electrode. CV curves were acquired at scan rates from 5 to 350 mV s^−1^ across a potential range of −0.6 to 0.6 V. Potential window was evaluated and set within range of 0 V to 0.6 V for the GCD experiments performed at varying current densities. EIS was performed at rms value of 10 mV sinusoidal signal, with a frequency range of 10^5^ Hz to 0.2 Hz. The specific capacitance (*C*_s_),^38^ were calculated using eqn (5) and (6) (ref. 39) are;5
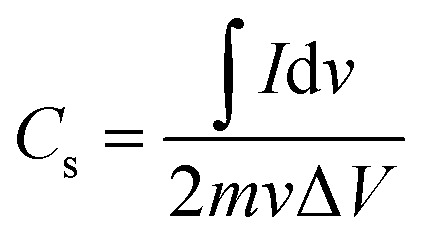
where “∫*I*d*v*” is an integrated area of curve, “*m*” is an active mass loading (g), “*v*” is a scan rate (V s^−1^), and “Δ*V*” is potential window (V).

Using the eqn (6), the *C*_s_ was calculated from GCD curves.^40^6
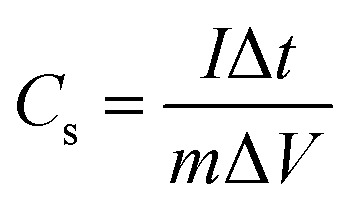
where “*I*_m_” is a current density (A g^−1^), “∫*V* d*t*” is area under the discharge curve, and “Δ*V*” is a potential window (V). Coulombic efficiency of ZCG nanocomposites were calculated using the eqn (7).^41^7
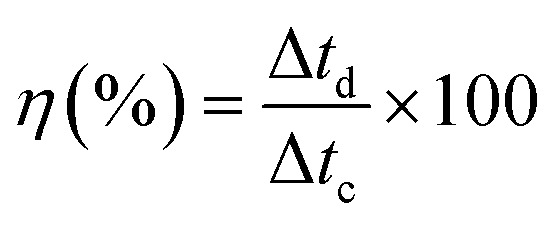
where “Δ*t*_d_” is discharging time and “Δ*t*_c_” is charging time.

Bode plots, on other hand, help to figure out how fast the electrode discharge at a phase angle 45°. This is called the relaxation time (*τ*).^42^ The relation between frequency response (knee frequency, *f*_o_) and the response time constant *τ* of supercapacitor can be expressed by eqn (8).^43^8
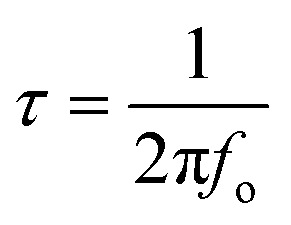


#### Coin cell calculations

Electrochemical measurements of ZCG10 device were performed using Gamry Reference 3000 under ambient conditions using 1 M KOH electrolyte. CV experiments were performed in a voltage window range of 0 to 1 V. Parameters for the coin cell device was evaluated using eqn (9)–(12).^44^ The specific capacitance (*C*_s_) from CV data was evaluated using eqn (9).9
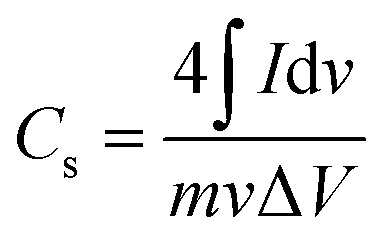


*C*_s_ from GCD data was calculated using eqn (10).10
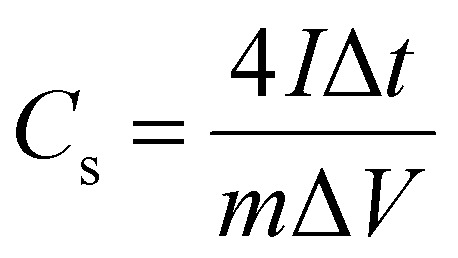


The power density (W kg^−1^) and energy density (W h kg^−1^) were calculated according to following eqn (11) and (12).11
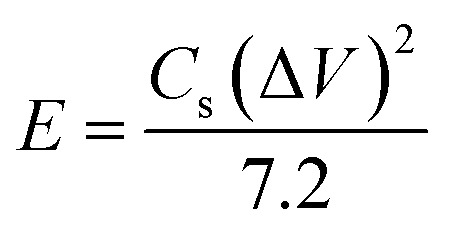
12
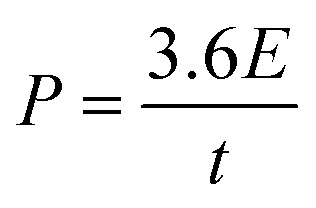


## Results and discussion

### XRD

**Fig. 4:**
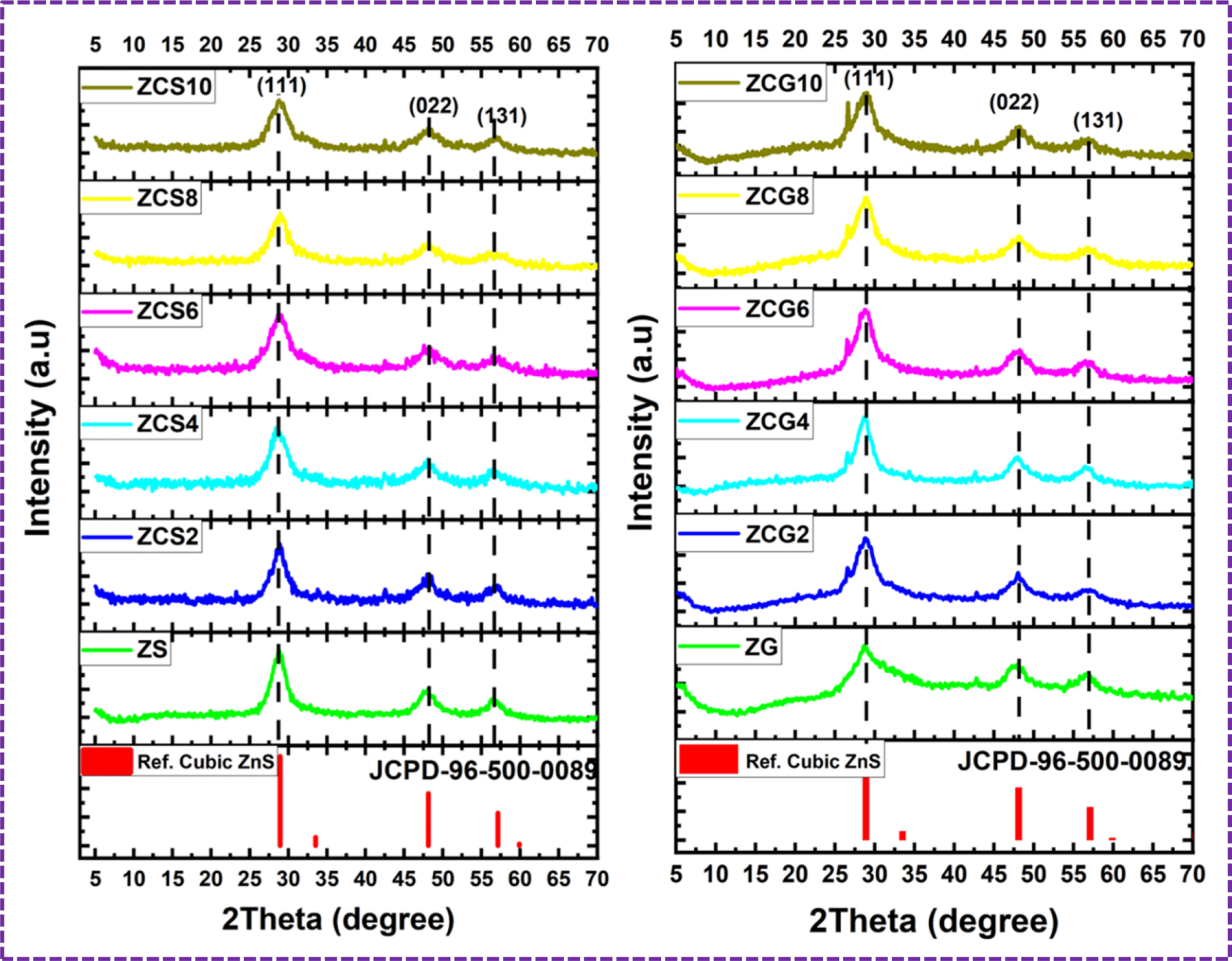
XRD patterns of (a) standard ZnS, ZS, ZCS2, ZCS4, ZCS6, ZCS8, ZCS10 and (b) standard ZnS, ZG. ZCG2, ZCG4, ZCG6, ZCG8, ZCG10.

XRD analysis was used to investigate crystallographic information of synthesized ZS, ZCS and ZCG (Fig. 4a and b). Cubic crystal system observed in accordance with the sphalerite ZnS. The diffraction peaks at 28.29°, 47.81°, and 57.16°, correspond to the (111), (022), and (131) crystal planes, similar to diffraction peaks of face-centered cubic sphalerite ZnS structure (JCPDS 96-500-0089). The crystallite size of prepared nanomaterials at high intensity peak are shown in (Table 1). Further, the un-doped ZnS powder had stronger peaks than the doped ones, and as the Cu-doping concertation increases, the peaks become broader. This suggests that the addition of Cu-doping lessens the nanocrystalline nature of the powder. The lack of extra peaks indicated that the prepared powders had no impurity phases.^45^

**Table 1:** XRD analysis of ZnS, ZCS2, ZCS4, ZCS6, ZCS8, ZCS10, ZG. ZCG2, ZCG4, ZCG6, ZCG8, and ZCG10

Sample	2*θ* (°)	FWHM (°)	Crystallite size (nm)
ZS	28.441	0.7085	11.57
ZCS2	28.8953	1.152	7.12
ZCS4	28.8984	2.9023	2.82
ZCS6	29.1472	3.03753	2.70
ZCS8	29.2441	3.11455	2.65
ZCS10	29.3217	3.16545	2.59
ZG	28.7209	1.152	7.12
ZCG2	28.9728	3.00881	2.73
ZCG4	28.9728	3.7574	2.18
ZCG6	29.0697	4.17287	1.97
ZCG8	29.1473	4.20025	1.96
ZCG10	29.1473	4.2994	1.91

However, due to the presence of graphene in the synthesized nanocomposite, no additional diffraction peaks were found. This might be attributed to the addition of minute quantities of graphene during the synthesis procedure.^46^

As the copper content increased, the as-prepared crystallite size of nanomaterials decreased from 7.12 nm to 2.65 nm. This could be variation in ionic radius between Cu^2+^ and Zn^2+^ ions. When Cu^2+^ ions are added to ZnS, gap between Cu^2+^ and Zn^2+^ ions in the ZnS lattice decreases because Cu^2+^ (73 pm) has a lower ionic radius than Zn^2+^ (74 pm). With copper doping of the ZCS particles, a reduction in crystallite size was observed due to the substitutional impurity behaviour of the dopant ion. A comparable trend was also documented in literature regarding ZnS thin films sprayed with Cu and dip-coated with Cu and Al-doping.^45,47^

The XRD patterns show that diffraction peaks shift towards higher 2*θ* angle due to Cu doping. This right shift usually causes the expansion of crystal lattice (Table 1). Consequently, the lattice parameter changes confirming the substitution of Cu^2+^ for Zn^2+^ in the crystal lattice.^48^

### FE-SEM

**Fig. 5:**
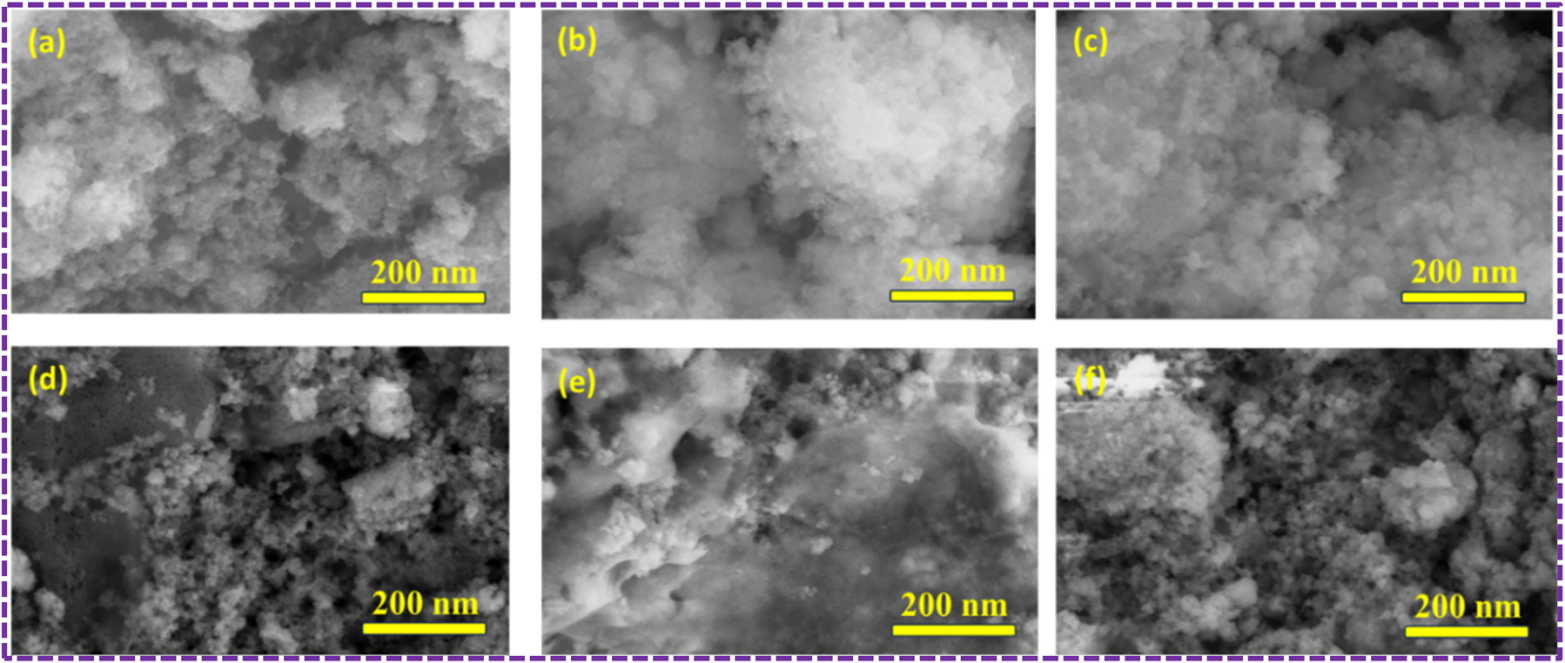
FESEM images of (a) ZS, (b) ZCS2, (c) ZCS4, (d) ZCS6, (e) ZCS8, and (f) ZCS10 at 200 nm.

The FE-SEM was used to analyse surface morphology of both pure and doped ZnS, ZCS and ZCG samples (Fig. 5). The FE-SEM demonstrates that ZCS samples exhibit a spherical morphology with a random size distribution for different Cu concentrations. The addition of Cu resulted in a decrease in particle size compared to pure ZnS nanoparticles. The particle size of as-prepared samples decreased from 55 nm to 22 nm with increase of Cu concentration. When Cu is doped into ZnS, Cu ions typically replace some Zn ions in the crystal lattice. Since Cu^2+^ ions (ionic radius 73 pm) are smaller than Zn^2+^ ions (ionic radius 74 pm), this substitution introduces lattice distortions or strain.^45,47^ Therefore, particle size of ZCS nanomaterials decrease with the increase of Cu concentration.

**Fig. 6:**
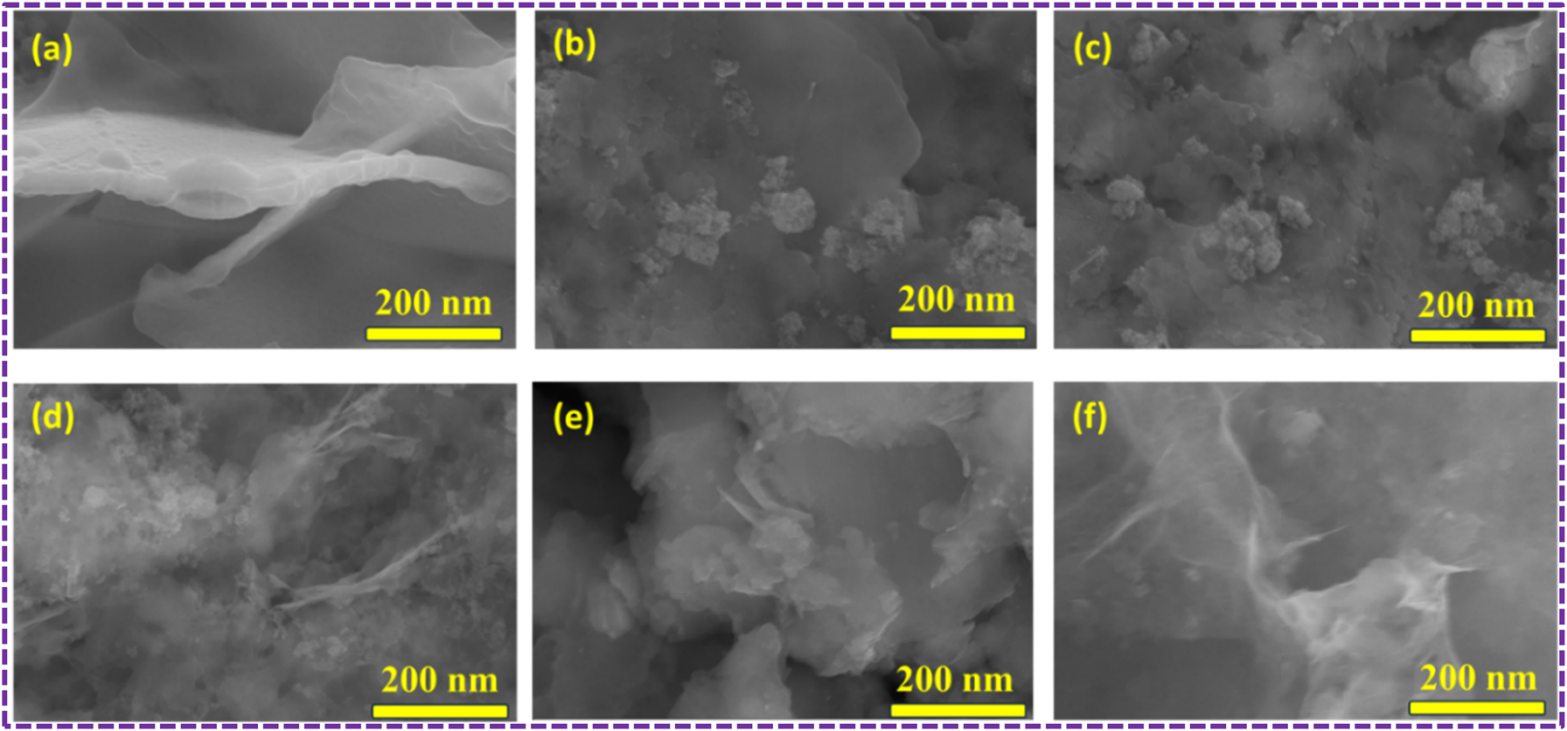
FESEM images of (a) ZG. (b) ZCG2, (c) ZCG4, (d) ZCG6, (e) ZCG8, and (f) ZCG10 at 200 nm.

The morphological study of the ZnS nanoparticles is uniformly attached to surface of graphene sheets (Fig. 6). The absence of graphene, no uniform distribution of ZnS were formed using the similar conditions used for synthesis of ZCG nanocomposites. These results clearly suggests that existence of graphene play an important role in formation of homogeneous ZnS aggregates. ZG and ZCG nanocomposites showed the sheets like structure.

### TGA/DSC

**Fig. 7:**
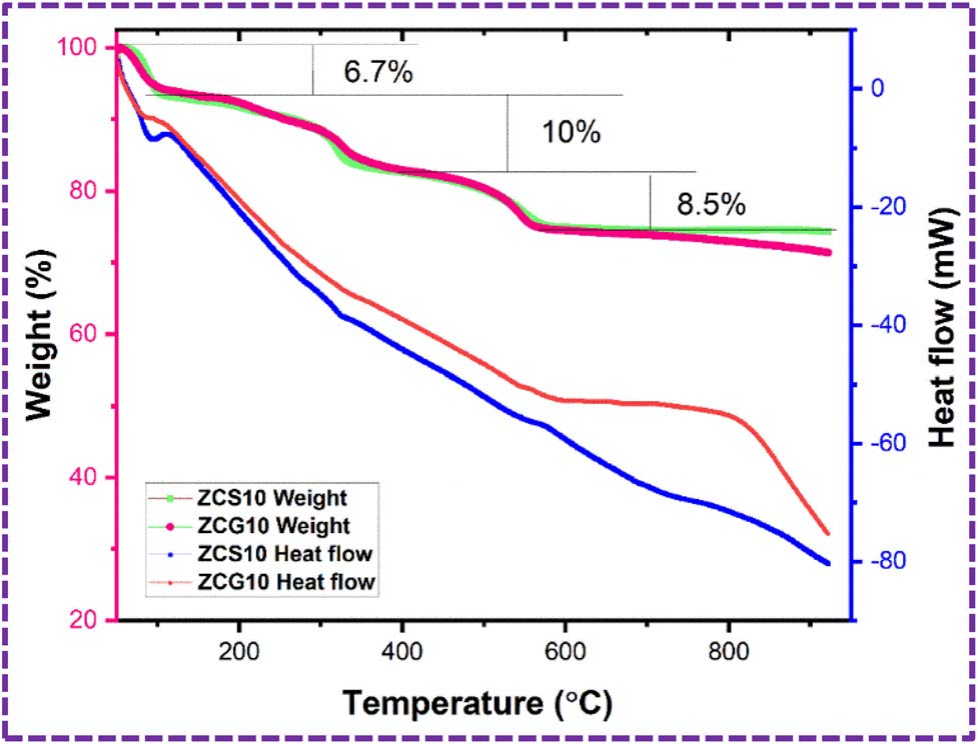
TGA (weight%) and DSC (heat flow) graph of ZCS10 and ZCG10.

TGA/DSC was performed to assess thermal stability of ZCS10 and ZCG10 nanocomposite. TGA curve of ZCS10 and ZCG10 exhibited three distinct weight loss segments between 74–100% and 71–100% representing only 26% and 29% of the total weight (Fig. 7). According to the analysis, the residual solvents evaporate at below 100 °C^49^ 6.7% of the sample's weight loss at temperature 71–105 °C was ascribed to the loss or evaporation of bound water molecules.^50^ The second weight loss, which occurs at temperature 105–345 °C, is 10% which attributed to structure changes of Cu-doped ZnS.^51^ The weight loss recorded in third region at 345–570 °C is 7.9% due to the thermal degradation of the carbon-chain framework^52^ and unstable components in the presence of samples.^49^ There is no weight loss after 570 °C. The final weight of the ZCS10 and ZCG10 nanocomposite is 74.3% and 71.3%. The weight loss in ZCG10 is more than ZCS10 which is attributed to the removal of carbon containing groups.^53^

The DSC associated with TGA of ZCS10 and ZCG10 nanocomposite exhibited heat flow modify with temperature and was used to determine crystallization and melting temperature. The crystallization temperatures of ZCS10 were determined at 326 °C and 576 °C and crystallization temperature of ZCG10 were at 347 °C and 557 °C.

### XPS

**Fig. 8:**
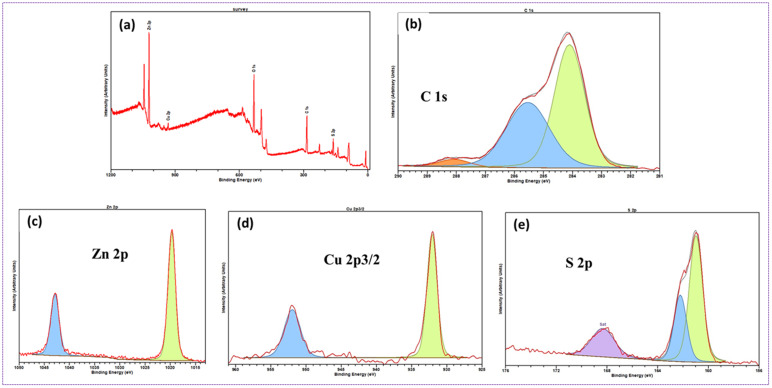
(a) XPS survey spectra of ZCS10 (b) spectra of C 1s (c) spectra of Zn 2p, (d) spectra of Cu 2p_3/2_, (e) spectra of S 2p.

Fig. 8 presents the XPS analysis of chemical compositions and metal oxidation states of ZCS10. The survey spectrum depicted in Fig. 8a presents the existence of C 1s, Zn 2p, Cu 2p_3/2_, and S 2p atoms in the ZCS10 composition. The C 1s exhibits three different signals, with the primary peak located at 284.1 eV attributed to sp^2^ and sp^3^ C–H/C–C carbon atoms. A secondary component is mostly attributed to C–O and C–N chemical environments at ∼286 eV peak. The peak component centred at 288.1 eV is finally attributed to C=O (Fig. 8b).^54^ The presence of Zn^2+^ oxidation states is indicated by binding energy of Zn 2p_3/2_ and Zn 2p_1/2_ at 1021 and 1043 eV (Fig. 8c). There is no visible peak shift after Cu doping.^55^ For the copper dopant, binding energies of Cu 2p_3/2_ and Cu 2p_1/2_ are 932 eV and 952 eV, as seen in Fig. 8d. These values can be attributed to either metallic or cationic Cu species according to previous studies.^55,56^ The spin–orbit interaction divides the S 2p level into two states with an energy difference of 1.2 eV. The S 2p_1/2_ and S 2p_3/2_ levels were detected at 162.2 eV and 161.0 eV, respectively, corresponding to ZnS. At 169.0 eV, which corresponds to SO_4_ of metal ions, oxidized sulfur was detected (Fig. 11e).^57^ All spectra confirm the chemical composition of the ZCS10.^58^ The XPS results further demonstrated the successful formation of composite.

**Fig. 9:**
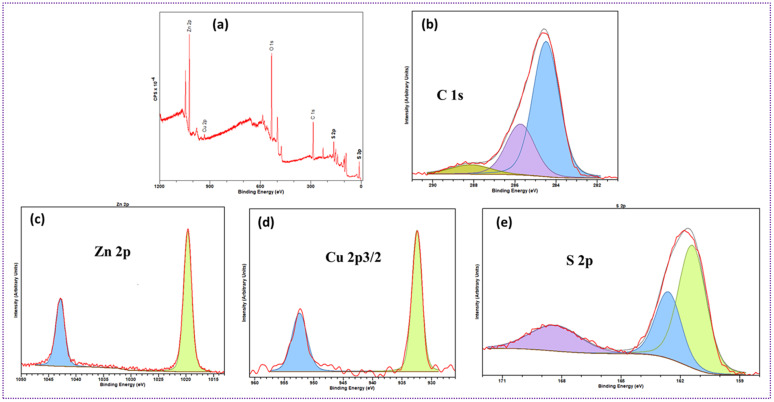
(a) XPS survey spectra of ZCG10 (b) spectra of C 1s (c) spectra of Zn 2p, (d) spectra of Cu 2p_3/2_, (e) spectra of S 2p.

Fig. 9 presents the XPS analysis of chemical compositions and metal oxidation states of ZCG10. The survey spectrum depicted in Fig. 9a presents the existence of C 1s, Zn 2p, Cu 2p_3/2_, and S 2p atoms in the ZCS10 composition. The C1s exhibits three different signals, with the primary peak located at 284.6 eV attributed to sp^2^ and sp^3^ C–H/C–C carbon atoms. A secondary component is mostly attributed to C–O and C–N chemical environments at ∼286 eV peak. The peak component cantered at 288.5 eV is finally attributed to C=O (Fig. 9b) The presence of Zn^2+^ oxidation states is indicated by binding energy of Zn 2p_3/2_ and Zn 2p_1/2_ at 1021 and 1043 eV (Fig. 9c). There is no visible peak shift after Cu doping.^55^ For the copper dopant, binding energies of Cu 2p_3/2_ and Cu 2p_1/2_ are 932 eV and 952 eV, as seen in Fig. 9d. These values can be attributed to either metallic or cationic Cu species according to previous studies.^55,56^ The S 2p_1/2_ and S 2p_3/2_ levels were detected at 162.5 eV, 161.7 eV and 161.7 eV, respectively, corresponding to ZnS. At 168.5 eV, which corresponds to SO_4_ of metal ions, oxidized sulfur was detected (Fig. 9e).^57^ All spectra confirm the chemical composition of the ZCS10.^58^ The XPS results further demonstrated the successful formation of composite.

### BET

The BET method is a standard approach used for measuring specific surface area, pore size and pore volume. The large surface area is a primary parameter for supercapacitors.^59^ The efficiency of supercapacitors can be improved by the enhanced transport of electrons and ions at the interface, as indicated by the BET analysis of ZCG10 nanocomposites with a large surface area. Additionally, the electrode storage capacity is increased by the presence of larger surface interfaces for charge (electrolyte ions) storage, which is facilitated by the large surface area.

The BET specific surface area, pore size and pore volume of the prepared sample was determined to be 104.3 m^2^ g^−1^, 1.99 nm and 0.052 cm^3^ g^−1^ for ZCG10 nanocomposite. The BET analysis indicates that ZCG10 nanocomposites with large surface area can enhance transport of electrons and ions at the interface, thereby improving the efficiency of supercapacitors.^60^

### Electrochemical measurements

The electrochemical performance of ZCG nanocomposites was thoroughly evaluated by CV and GCD measurements at room temperature in three electrode system. When ZCG nanocomposites prepared for supercapacitor applications in 1 M KOH electrolyte, the electrochemical reactions can involve various processes including ion adsorption/desorption and redox reactions. Here's a representation of the electrochemical reactions:

Adsorption of K^+^ ions onto graphene surface:

13 Graphene + KOH → graphene_K + OH^−^

Adsorption of OH^−^ ions onto graphene surface:

14 Graphene + OH^−^ → graphene_OH

Redox reactions involving Cu dopants and ZnS during charging and discharging:^61^

15 Cu^+^ + 2OH^−^ → Cu(OH)_2_ + 2e^−^

The non-faradaic process may be due to the development of double layer at electrode/electrolyte interfaces during disinsertion/insertion of positive ions (K^+^) on ZnS nanoparticles.

16 ZnS(surface) + K^+^ + e^−^(ZnS–K^+^)surface

The faradaic reaction process is also detected in ZnS causes slight deviation of rectangular shape in CV curve at all the scan rates. The possible faradaic process can be explained as follows,^62,63^

17 ZnS + OH^−^ ⇌ ZnSOH + e^−^

18 ZnSOH + OH^−^ ⇌ H_2_O + ZnSO + e^−^

**Fig. 10:**
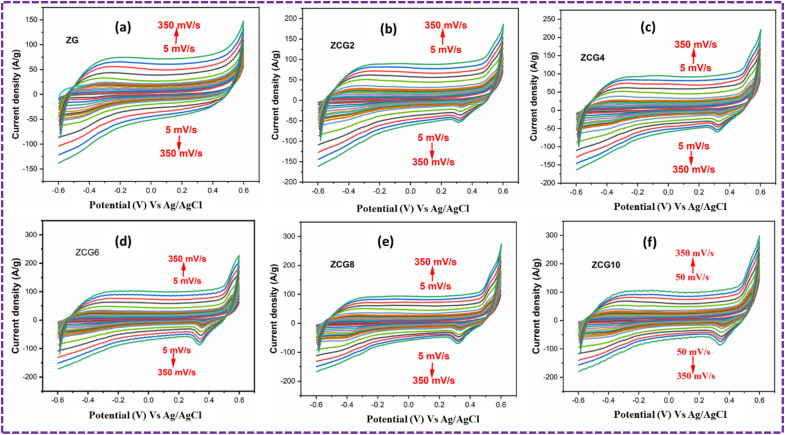
CV curves of all samples at 5–350 mV s^−1^ scan rates in three electrode system. (a) ZG, (b) ZCG2, (c) ZCG4, (d) ZCG6, (e) ZCG8 and (f) ZCG10.

**Fig. 11:**
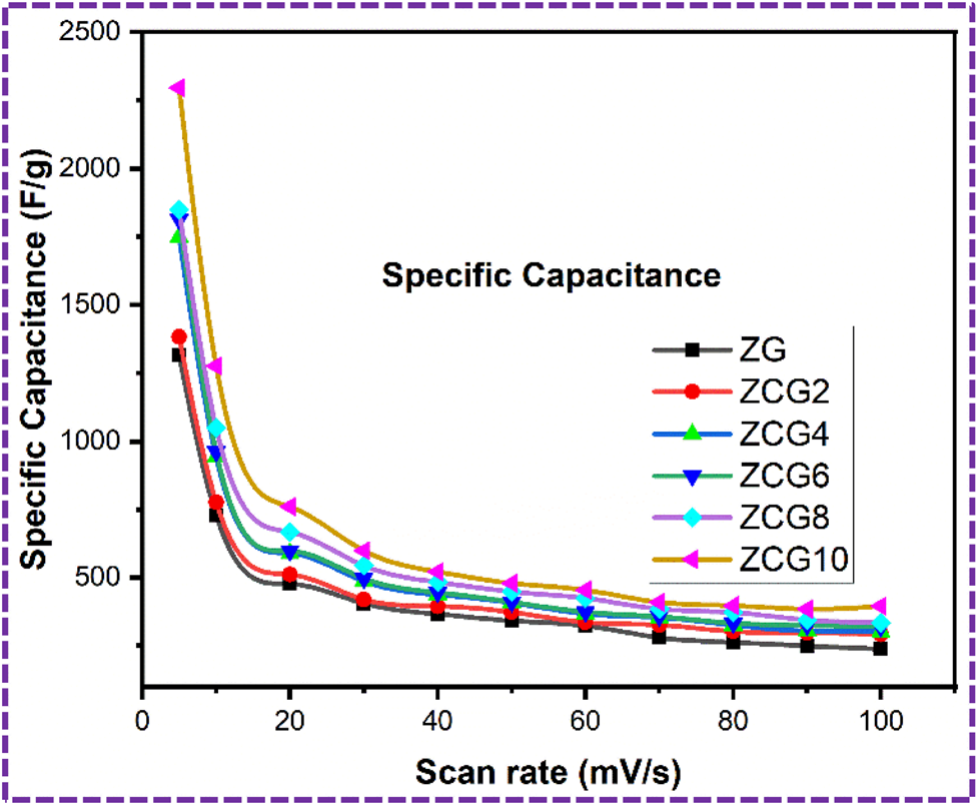
Comparison of specific capacitance graph by CV.

A three-electrode system was used to investigate electrochemical measurements. Fig. 10 displays the comparative CV curves of ZG, ZCG2, ZCG4, ZCG6, ZCG8, and ZCG10 nanocomposites. The curves were obtained using a constant scan rate of 5–350 mV s^−1^ and within the potential window −0.6 to 0.6, CV curves demonstrate that the Cu-doped ZnS nanocomposites exhibit larger areas under their CV curves compared to the undoped ZnS, therefore emphasizing the significant contribution of Cu in enhancing the electrochemical performance. The Cs of the ZCG nanocomposites curves increases with the increase of Cu concentration.^16^ The CV curves exhibit a capacitive behaviour with nearly a rectangular shape, denoting a reversible system.^64^ The calculated values are given in Table 2. The enhanced electrochemical characteristics of ZCG10 can be attributed to presence of redox-peaks and electrochemically active nature of the doped electrode materials compared to the ZG nanocomposite.^16^ The redox peaks become higher with increase of scan rate. The specific capacitance of ZCG10 nanocomposite is higher than other nanocomposites (Fig. 11). Furthermore, the CV curves exhibited excellent stability even at a rapid scan rate of 350 mV s^−1^, indicating a fast electrochemical response.

**Table 2:** Cyclic voltammetry measurements of ZG, ZCG2, ZCG4, ZCG6, ZCG8 and ZCG10 Electrodes

Samples	Electrolyte (M KOH)	Scan rate (mV s^−1^)	Specific capacitance (F g^−1^)	Efficiency (%)
ZG	1	5	1312	99
ZCG2	1	5	1381	94.7
ZCG4	1	5	1749	89.1
ZCG6	1	5	1813	88.9
ZCG8	1	5	1848	88.8
ZCG10	1	5	2295	85.8

**Fig. 12:**
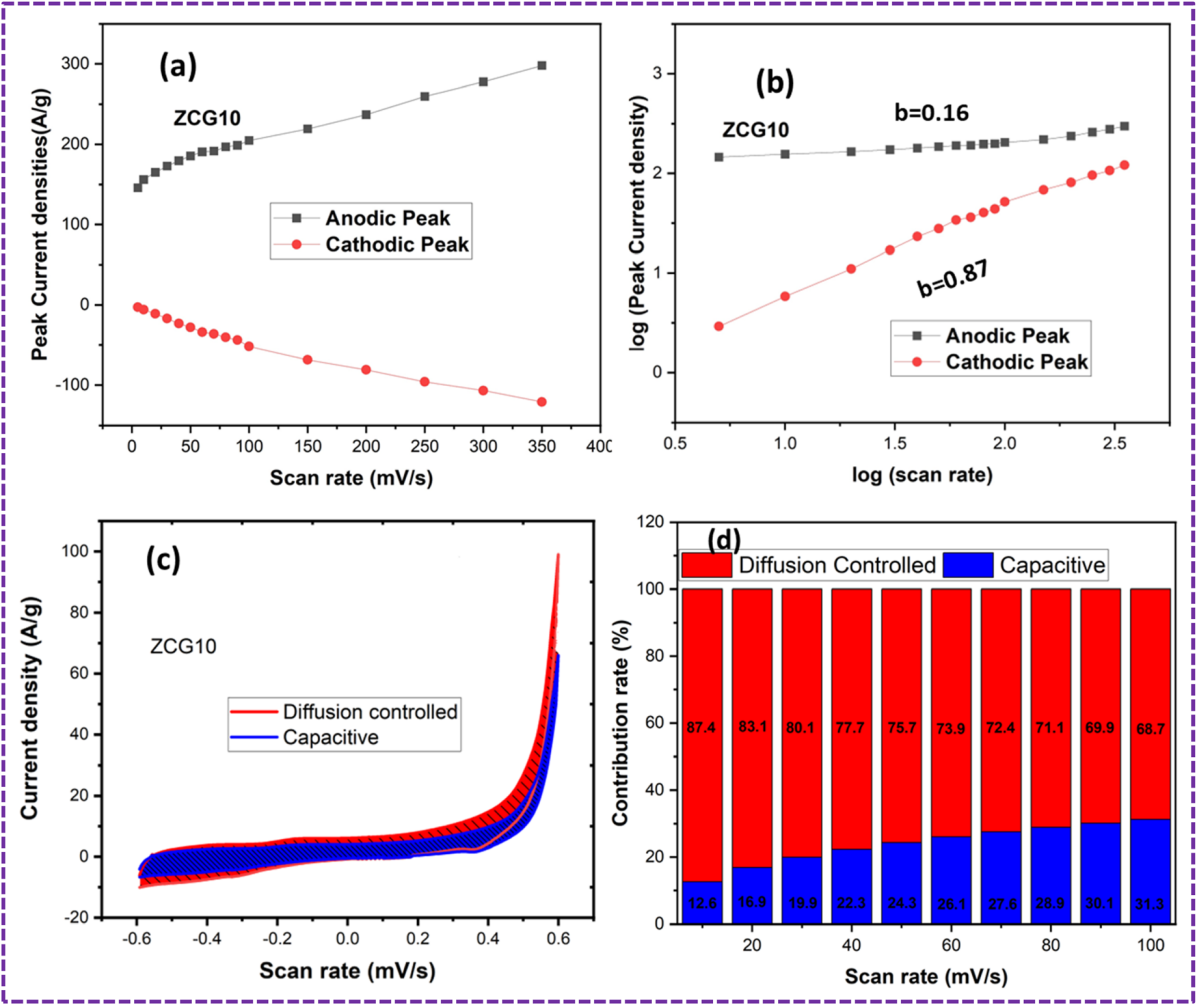
Charge storage mechanism of ZCG10 nanocomposite: (a) graph between scan rate (mV s^−1^) and peak current density (A g^−1^) (b) graph between log(scan rate) and log(peak current density) (c) graph of diffusion-controlled and capacitive contribution at 20 mV s^−1^ and (d) percentage of diffusion-controlled and capacitive contribution at different scan rates.

The kinetics of the charge storage mechanism of ZCG10 nanocomposites electrodes were analysed from CV curves (Fig. 12). The graph between the scan rate (mV s^−1^) and peak current densities (A g^−1^) is shown (Fig. 12a) which corresponds to a linear relationship and indicates that the charge storage is hybrid (Fig. 12b). for further confirmation of this hypothesis, we used the slopes of the log(*i*) *vs.* log(*v*) plot to derive *b* values (Fig. 12d) *via* equations.^65^

19 *i* = *av*^*b*^

20 log *i* = log *a* + *b* log *v*

The ZCG electrode has a EDLC behavior, as indicated by *b* values in the range. It is known that diffusion-controlled mechanism occurs when *b* = 0.5, while capacitive contribution occurs when *b* = 1. The percentage of charge contributed by diffusion and capacitance may also be estimated by equations.^65,66^

Eqn (15) has been used to evaluate percentage of diffusion and capacitive charge contribution.^65^

21 *i*(*V*) = *k*_1_*v* + *k*_2_*v*^1/2^

where “*i*” is current density, “*k*_1_“and “*k*_2_“can be determined by plotting the fitting line of the above equation and “*ν*” is scan rate. The linearity of plots (Fig. 12b) suggest that electrochemical redox reactions take place on the electrode surface *via* diffusion-controlled process. The capacitive contribution percentage continues to improve as the scan rate becomes higher with the fast electrochemical activities (Fig. 12c and d). This is because the hydroxide ions have less time to diffuse into electrode materials at higher scan rates and can only approach outer surface-active sites.^65^ The CV measurements of ZCG nanocomposites are in Table 3.

**Table 3:** GCD measurements of ZG, ZCG2, ZCG4, ZCG6, ZCG8 and ZCG10 electrodes

Samples	Electrolyte (M KOH)	Specific capacitance (F g^−1^)	Energy density (W h kg^−1^)	Power density (W kg^−1^)	Capacitive retention (%)	Columbic efficiency (%)
ZG	1	473.4	23.66	12 657.3	98 after 1000 cycles	96 after 1000 cycles
ZCG2	1	507.4	25.36	14 355.59	94 after 1000 cycles	99 after 1000 cycles
ZCG4	1	517.3	25.85	18 109.53	91 after 1000 cycles	99 after 1000 cycles
ZCG6	1	528.2	26.40	25 077.65	90 after 1000 cycles	93 after 1000 cycles
ZCG8	1	589.4	29.46	47 989.43	90 after 1000 cycles	93 after 1000 cycles
ZCG10	1	669.9	33.48	62 782.19	89 after 1000 cycles	93 after 1000 cycles

**Fig. 13:**
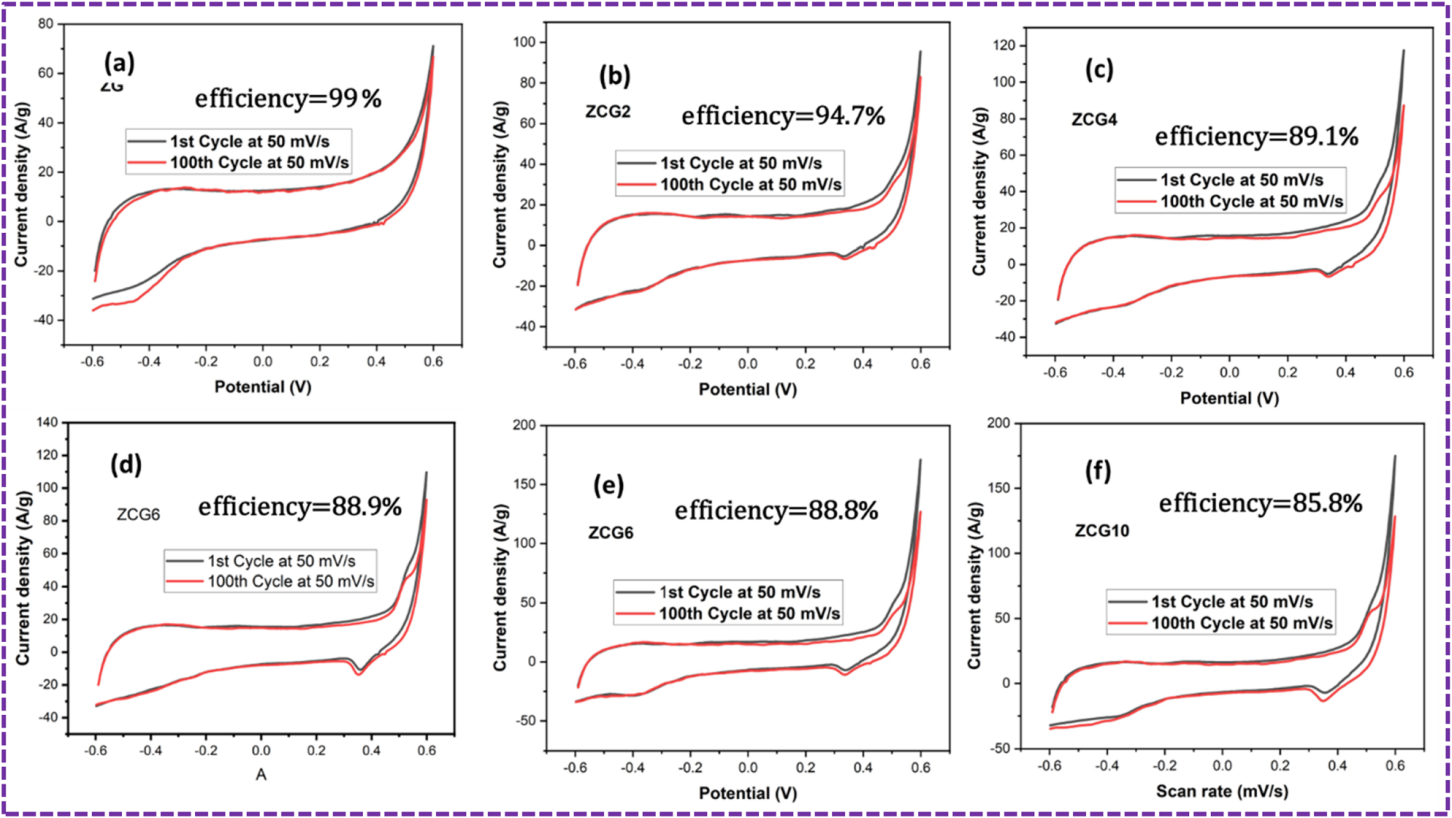
Graph of efficiency comparison, (a) ZG, (b) ZCG2, (c) ZCG4, (d) ZCG6, (e) ZCG8 and (f) ZCG10.

Fig. 13a shows the 1st and 100th cycle of CV curves at 50 mV s^−1^ which is used to calculated the efficiency of nanocomposites.

**Fig. 14:**
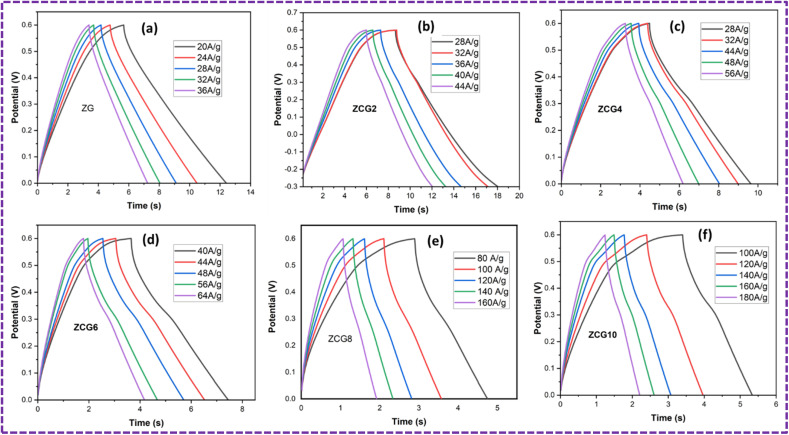
GCD at different current densities of (a) ZG, (b) ZCG2, (c) ZCG4, (d) ZCG6, (e) ZCG8 and (f) ZCG10.

GCD curves of optimal ZCG electrodes with various current densities were also evaluated at potential range 0f 0.0–0.6 V in Fig. 14. It was evident that at various current densities, the electrodes of nanocomposites exhibited almost similar curves at different current densities. GCD curves have a isosceles triangular shape,^67^ indicating good electrochemically capacitive behaviour with less faradaic reaction.^68,69^ Furthermore, IR drop at different current densities is quite small. This confirms the capacitive characteristics of the system, which demonstrate excellent electrochemical reversibility. The electrodes specific capacitance was determined using eqn (6). The ZCG10 electrode has the greatest specific capacitance compared to all other electrodes under the given current densities. The findings suggest that charge transfer occurs at a rapid and effective rate, and that the ZCG10 electrode had retain its high capacitance at high current density.

**Fig. 15:**
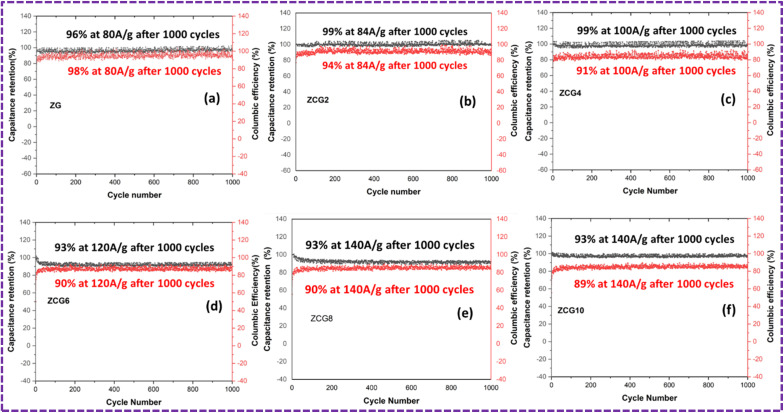
Cyclic performance of (a) ZG, (b) ZCG2, (c) ZCG4, (d) ZCG6, (e) ZCG8 and (f) ZCG10 after 1000 cycles.

The cyclic stability of ZCG electrodes was measured at different current densities. After 1000 cycles, coulombic efficiency and capacitance retention of ZCG10 electrode was 89% and 93% Fig. 15. The GCD measurements are in Table 3.

**Fig. 16:**
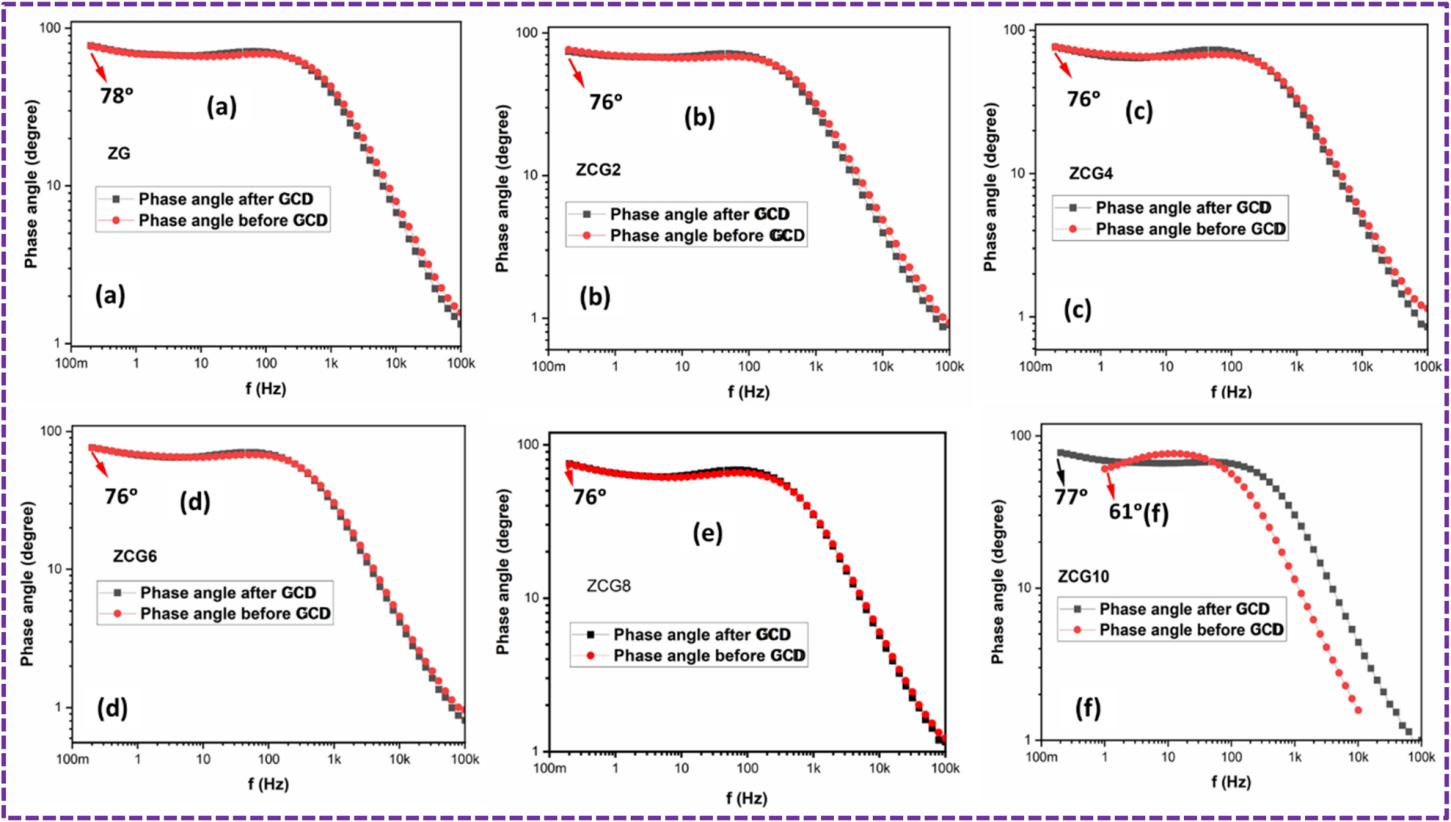
Graph between frequency and phase angle of, (a) ZG, (b) ZCG2, (c) ZCG4, (d) ZCG6, (e) ZCG8 and (f) ZCG10 before and after GCD.

Fig. 16 illustrates the relationship between frequency and phase angle using a Bode plot, whereby the knee frequency is seen at a phase angle of 45° (ref. 70) which is 790.9, 510.5, 524.6, 510.5, 518.3, and 503.5 s^−1^. The phase angle and response time mentioned in Table 4.

**Table 4:** EIS measurements of ZG, ZCG2, ZCG4, ZCG6, ZCG8 and ZCG10 Electrodes

Samples	Phase angle (degree)	Knee frequency (Hz)	Response time constant (s)
ZG	78	790.9	0.0002
ZCG2	76	510.5	0.00031
ZCG4	76	524.6	0.00031
ZCG6	76	510.5	0.00031
ZCG8	75	518.3	0.00031
ZCG10	77	503.5	0.00031

**Fig. 17:**
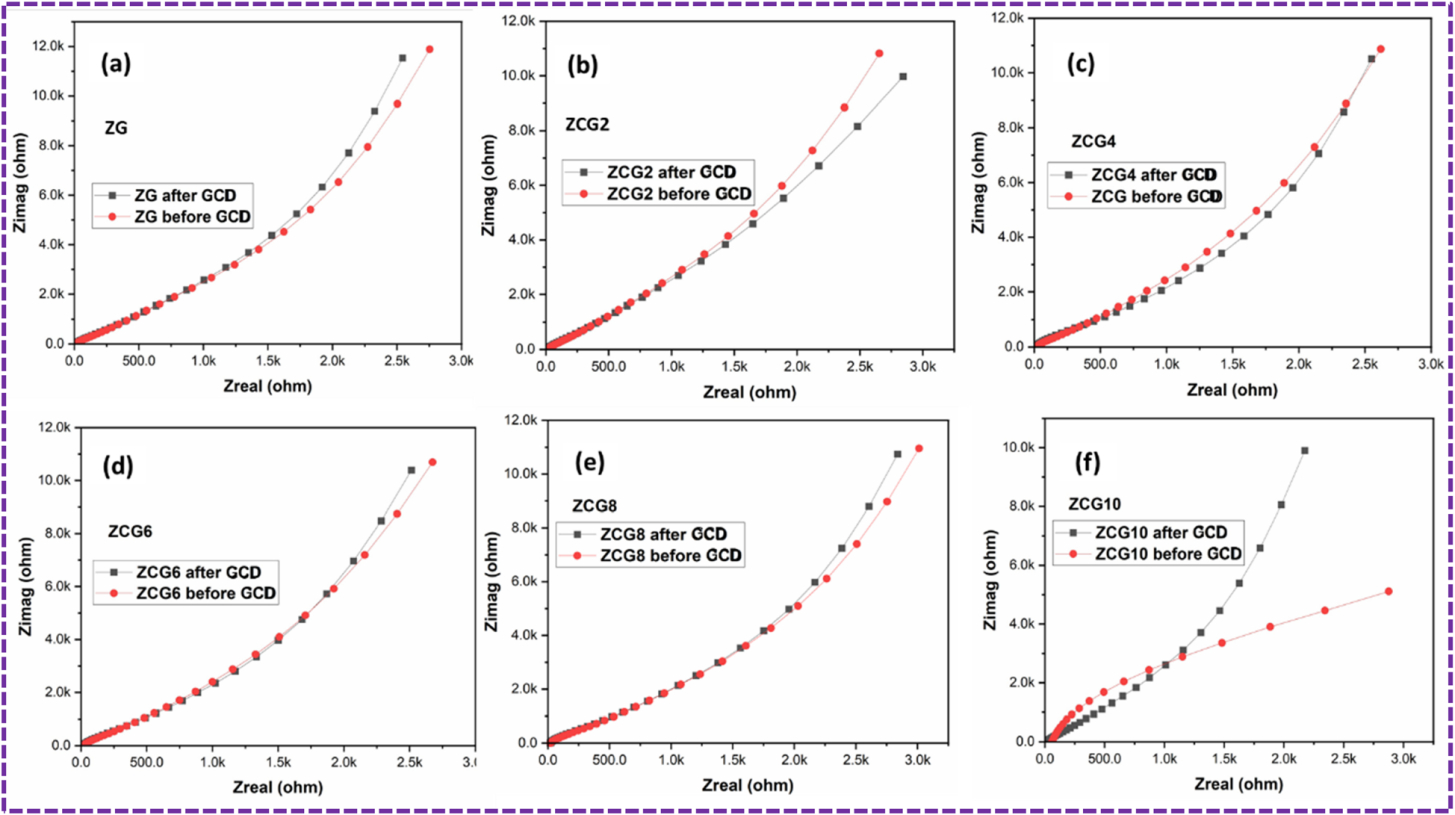
Nyquist plot of (a) ZG, (b) ZCG2, (c) ZCG4, (d) ZCG6, (e) ZCG8 and (f) ZCG10 after 1000 cycles before and after GCD.

The Nyquist plot of ZCG electrodes were plotted (Fig. 17) represent the depressed semicircles with inclined lines were displayed in the high-frequency and low-frequency region, respectively (Fig. 17). Curiously, all of the electrodes had comparable EIS having negligible semicircle region, which is known as charge transfer resistance (*R*_ct_), which could be indicates a low faradaic rate of charge–discharge.^71^

**Fig. 18:**
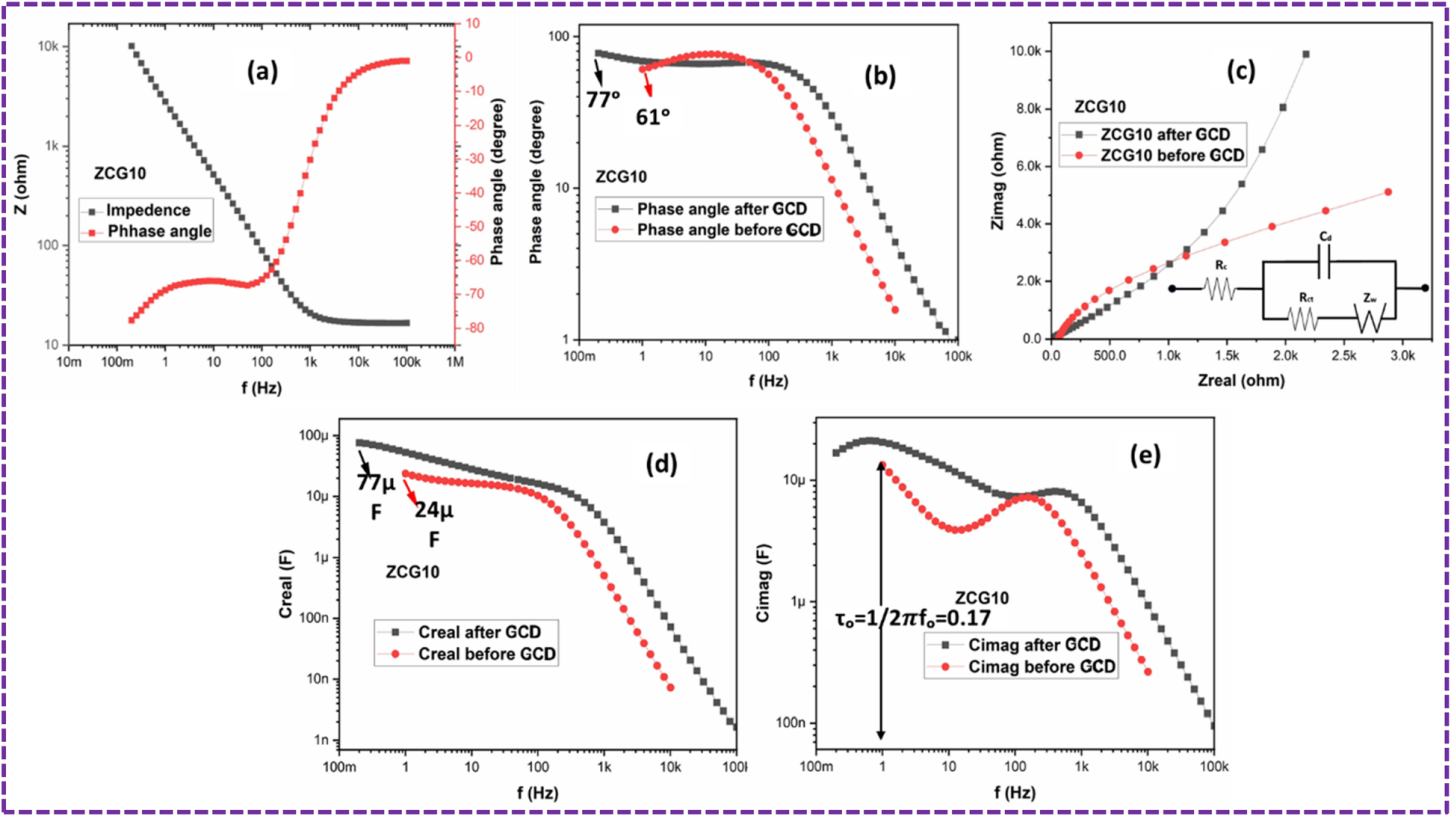
(a) Bode plot of ZCG10 electrode (b) plot of frequency and phase angle (c) Nyquist plot of ZCG10 electrode (d) real capacitance and (e) imaginary capacitance as a function of frequency for supercapacitor in ZCG10.

Fig. 18 shows the EIS study of ZCG10 nanocomposite. Bode plot illustrating the frequency response of the phase angle and impedance of the ZCG10 nanocomposite (Fig. 18a). (Fig. 18b) illustrates the relationship between frequency and phase angle using a Bode plot, whereby the knee frequency is seen at a phase angle of 45° (ref. 70) which is 503.5 s^−1^.

Fig. 18c shows the Nyquist plots of the ZCG10 nanocomposite electrode before and after the GCD cyclic stability. In both irradiated and dark cases, Nyquist plots exhibit a straight slope line at low frequencies and a prominent semicircle at high frequencies. The charge-transfer resistance at the electrode interface is represented by the diameters of the semicircles, while diffusion process of reactive species is represented by the straight sloping line. The model fitting was done by the Gamry Echem Analyst (inset in Fig. 18c). The term “*R*_s_” refers to the resistance of the electrolyte, which includes the resistances related to contact and charge transfer at counter electrode/electrolyte interface.^72^*R*_ct_ refers to the resistance produced during electron transport. The fitting results showed the calculated values of *R*_e_ and *R*_ct_ for the electrodes. The ZCG10 electrode exhibits respective *R*_e_ and *R*_ct_ which are 60.2 Ω and 7 kΩ. The constant phase element (CPE) is a term used to describe the double layer capacitance. The Warburg impedance (*Z*_w_) represents the diffusion of reactive material at electrode surface and is depicted as a straight line in Nyquist plots.^65,73^

Additionally, ZCG electrodes exhibited high specific capacitance which is calculated by *C*_real_ (F) over frequency (Hz) (Fig. 18d). The response time of supercapacitors are calculated by *C*_imag_ (F) over frequency (Hz) (Fig. 18e). Fig. 18d in the electrolyte shows a higher specific capacitance before and after GCD which is 24 and 77 μF. Fig. 18e shows that the response time of the supercapacitors which is 0.17 s calculated from frequency and *C*_imag_ graph in the KOH electrolyte solution which ascribed to the charge transfer resistance.^71^Table 5 represent the comparative study of ZCG10 electrode with previous published literature.

**Table 5:** Comparative study of ZCG10 electrode with previous published literature

Sr#	Material	Synthesis method	Electrolyte	Scan rate/current density	Specific capacitance	Potential window (V)	Cyclic stability	Ref.
1	ZnS/graphene	Hydrothermal	2 M KOH	5 mV s−1	315.1 F g−1	−0.2 to 0.3	63.2% after 1000 cycles	74
2	Cu-doped ZnS	Single-step glycol-assisted process	3 M KOH	1 A g−1	468 F g−1	0.0 to 0.5	90.5% after 5000 cycles	16
3	ZnS:Cu	Solvothermal	3 M KOH	1 A g−1	545 F g−1	0 to 0.4	87.4% after 5000 cycles	75
	Cu doped ZnS/G	Coprecipitation	1 M KOH	100 A g^−1^	743 F g^−1^	0 to 0.6	89% after 1000 cycles	This work

### ZCG10 symmetric coin cell

**Fig. 19:**
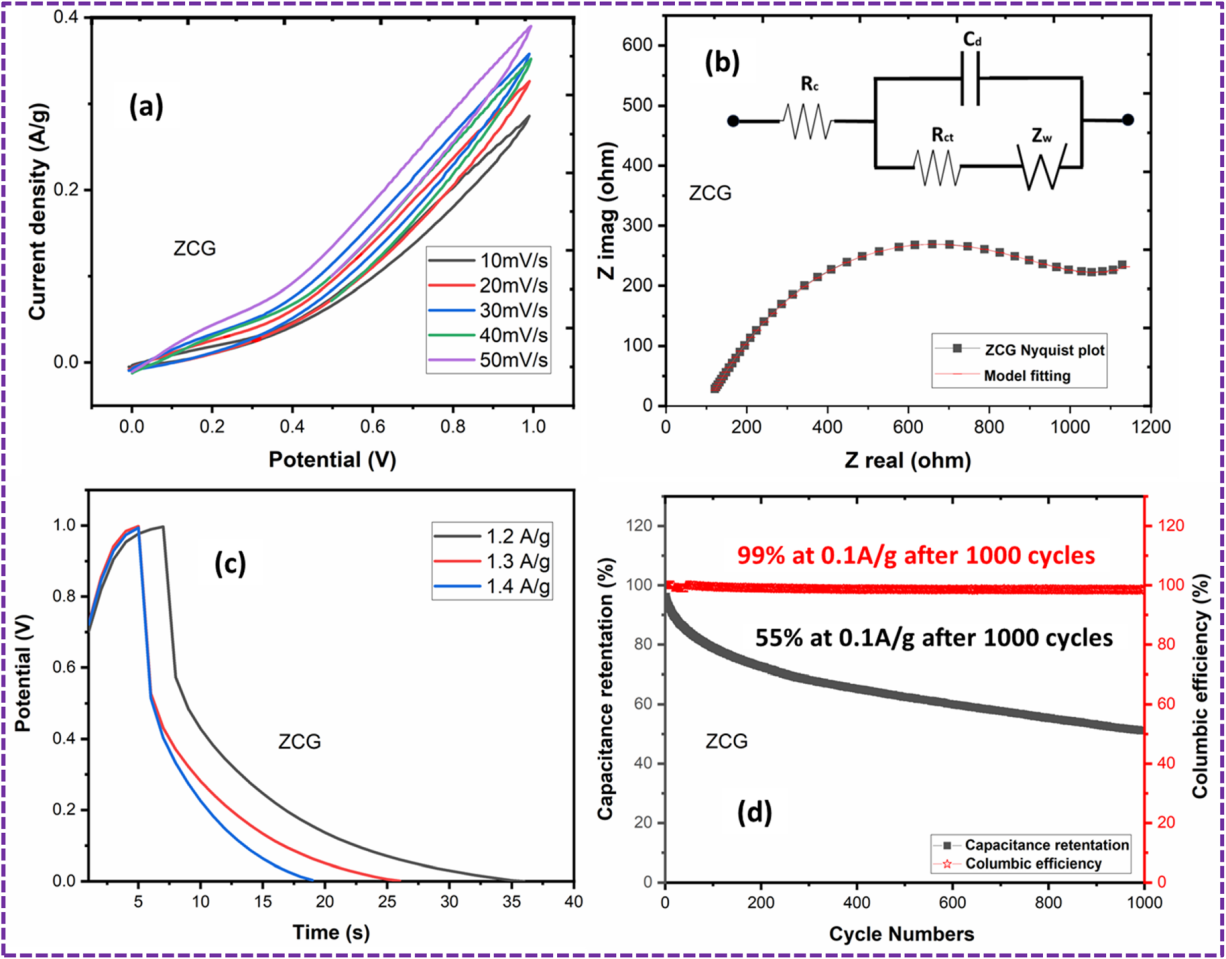
(a) CV curve of ZCG10 coin cell (b) Nyquist plot of ZCG10 coin cell (inset model fitting) (c) GCD curves of ZCG10 coin cell (d) cyclic performance o ZCG10 coin cell after 1000 cycles.

To evaluate the practical application of ZCG10 electrodes, a coin cell was fabricated utilizing 1 M KOH aqueous electrolyte. Fig. 19 displays the CV curves of coin cell obtained at various potential windows. The results demonstrate that the greatest operational potential window achievable is 1 V. The CVs of the supercapacitor obtained using varying scan rates ranging from 10 to 50 mV s^−1^ (Fig. 19a). The CV curves exhibiting symmetric and rectangular shape with little lean, indicate typical capacitive characteristics, similar to the results obtained from three-electrode experiment. The specific capacitance of ZCG10 coin cell is 72 F g^−1^ calculated using the eqn (9).

Fig. 19b depicts the Nyquist plot of ZCG10 coin cell.

The GCD curves of ZCG10 coin cell in Fig. 19c. The fabricated cell exhibited a specific capacitance of 130.8 F g^−1^, an energy density of 18.06 W h kg^−1^ and a power density of 2400 W kg^−1^ when operating at a current density of 0.12 A g^−1^ calculated by eqn (10)–(12).^76^

The cycle stability of the ZCG10 coin cell after 1000 cycles was evaluated at a current density of 0.1 A g^−1^ (Fig. 19d). The capacitance retention dropped to about 75% after 200 cycles, but the tendency of the capacitance retention drops gradually in subsequent cycles. Finally, capacitance retention of the cell remained at 55% after 1000 cycles. The coulombic efficiency is about 99% at 0.1 A g^−1^ after 1000 cycles.^58^Table 6 represents the electrochemical measurements of ZCG10 coin cell. Table 6 represents the electrochemical measurements of ZCG10 coin cell. Table 7 represent the comparative study of ZCG10 coin cell with previous published literature.

**Table 6:** Electrochemical measurements of ZCG10 coin cell

Current density (A g^−1^)	Specific capacitance (F g^−1^)	Energy density (W h kg^−1^)	Power density (W kg^−1^)	Capacitive retention (%)	Columbic efficiency (%)
1.2	130.8	18.06	2400	55 after 1000 cycles	99 after 1000 cycles

**Table 7:** Comparative study of ZCG10 device with previous published literature

Material	Electrolyte	Current density	Specific capacitance	Potential window (V)	Energy density (W h kg^−1^)	Power density (W kg^−1^)	Ref.
rGO/ZnO/PDAN	PVA/KOH	0.6 A g^−1^	40 F g^−1^	0 to 1.2	12.66	933	77
ZnS/g-C_3_N_4_	6 M KOH	0.5 A g^−1^	92.8 F g^−1^	0 to 0.9	10.4	187.3	78
ZnS/NiO	1 M KOH	0.8 A g^−1^	44.43 F g^−1^	0 to 1	5.52	1600	63
Cu doped ZnS/G	1 M KOH	1.2 A g^−1^	130.78 F g^−1^	0 to 1	18	2400	This work

## Conclusions

In this work, a Cu doped ZnS (ZCS) was synthesized *via* chemical coprecipitation method and graphene (G) is synthesized *via* electrochemical exfoliation method. After that ZCS/G (ZCG) nanocomposite prepared *via* ultrasonication method. Using appropriate analytical characterization techniques, the successful formation of ZCS, G and ZCG nanocomposite electrode was confirmed. XRD analysis confirmed the cubic phase of pure ZnS and ZCS. FE-SEM observation demonstrated that ZnS, ZCS and ZCG nanocomposite contains nanomaterials. The ZnS/NiO electrode exhibited remarkable electrochemical performance as an electrode material. The ZCG10 electrode exhibited an ultrahigh specific capacitance of 2295.04 F g^−1^ at a relatively low scan rate of 5 mV s^−1^ in a 1 M KOH solution from CV and 743 F g^−1^ at 100 A g^−1^ from GCD. After 1000 cycles, the ZCG10 nanomaterial demonstrated an enhanced and praiseworthy stability performance of 89%. Furthermore, the ZCG10 device exhibited a specific capacitance, energy density, power density of 130.8 F g^−1^, 18 W h kg^−1^, 2400 W kg^−1^ at current density of 1.2 A g^−1^ in a 1 M KOH solution from GCD. After 1000 cycles, the ZCG10 nanomaterial demonstrated stability performance of 55%. This ZCG nanocomposite is a potential candidate for high performance of supercapacitor devices and hybrid material design is an effective strategy for developing high-performance electrode materials.

## Author contributions

The manuscript was written through contributions of all authors. All authors have given approval to the final version of the manuscript.

## Conflicts of interest

There are no conflicts to declare.

## Supplementary Material

RA-015-D5RA01710F-s001

## Data Availability

The data supporting this article have been included as part of the ESI.†
