# Supplementary material for: Fabrication and electrochemical study of copper doped zinc sulfide/graphene nanocomposites for supercapacitors
Source: RSC Adv. 2025 Jul 11;15(30):24331–49. doi: 10.1039/d5ra01710f (PMC12246917; doi:10.1039/d5ra01710f)
Supplement: RA-015-D5RA01710F-s001 [file RA-015-D5RA01710F-s001.pdf]

Supplementary Information (SI) for RSC Advances.
This journal is © The Royal Society of Chemistry 2025

Supporting Information

Fabrication and Electrochemical Study of Copper doped Zinc Sulfide/Graphene Nanocomposites for Supercapacitors

*Shumaila Saleem[a,b], Sadia Khalid[*b], Aalia Nazir[a], Yaqoob Khan[b], Imtiaz Ahmad[b,c], Rameesha[b,d]*

[a] Institute of Physics, The Islamia University of Bahawalpur, 63100, Pakistan

[b] Nanosciences & Technology Department, National Centre for Physics, Quaid-e-Azam University Campus, Shahdra Valley Road, Islamabad, 45320, Pakistan

[c] Department of Physics, University of Science and Technology (UET), Lahore, 54890, Pakistan

[d] Department of Physics, Faculty of Science, University of Sialkot, Sialkot, 50700, Pakistan

Correspondence: sadiabzu@gmail.com, sadia.khalid@ncp.edu.pk

Energy Dispersive X-ray (EDX)

Figure S1 shows the presence of Zn, Cu, and S in ZCS2, ZCS4, ZCS6, ZCS8, and ZCS10 nanomaterials. Figure S1 qualitatively indicated the concentration of Cu increase from ZCS2 to ZCS10 nanomaterials.

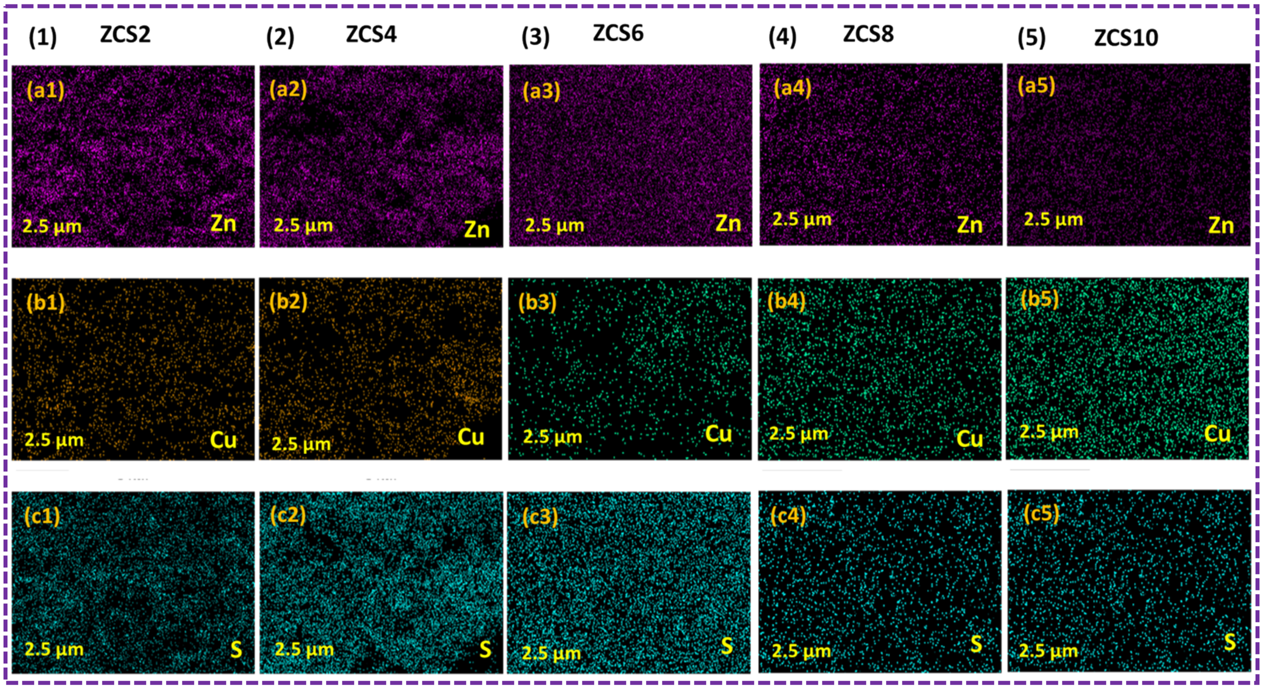

Figure S1. Elemental distribution of (1) ZCS2, (2) ZCS4, (3) ZCS6, (4) ZCS8 and (5) ZCS10 nanomaterials.

Figure S2 showed the results of elemental mapping which indicate the presence of Zn, S, Cu, and C, suggesting that ZCG nanoparticles are present. Throughout the nanocomposite, Zn, S, Cu, and C atoms were found to be evenly dispersed.

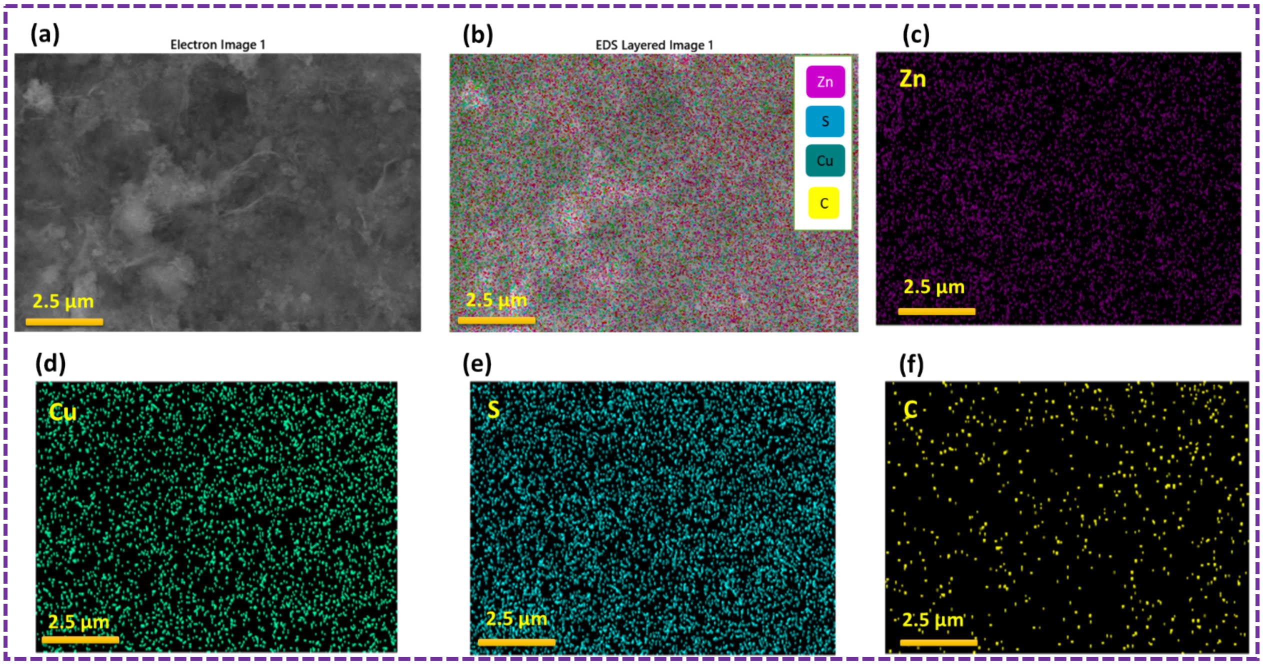

Figure S2. (a) SEM image of ZCG10; (b) EDX mapping and elemental analysis of ZCG10 (c) Zn, (d) Cu, (e) S, and (f) C.

Diffuse reflectance spectroscopy (DRS)

The band gap energies of ZnS, ZCS, and ZCG nanocomposite were evaluated using the DRS spectra (Figure S3). This was achieved by plotting the photon energy (eV) and extrapolating the linear portion of the curve of F (R). $E^2 = 0$ against the square of Kubelka–Munk function F (R). The calculated band gaps for ZnS, ZCS2, ZCS4, ZCS6, ZCS8, ZCS10, are 3.59, 3.58, 3.57, 3.56, 3.52 and 3.46 eV, which indicates that the band gap of nanomaterial decreases with the increase of Cu doping. There are several factors that may cause decrease in optical band gap i.e., particle size, lattice parameter, microstrain, and dopant concentration. This decrement also indicates the formation of new energy levels near the conduction band due to substitution of Cu^{2+} impurities. Consequently, the Fermi level also changes that may alter the electronic and electrical properties [1]

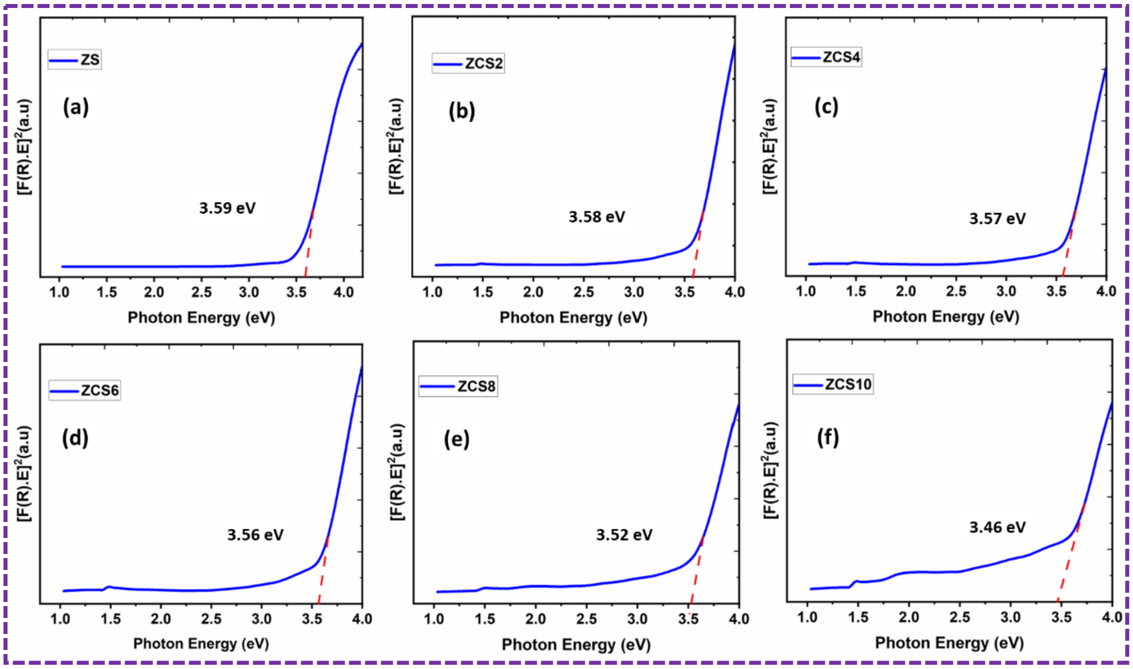

Figure S3. UV-vis DRS of (a) ZS, (b) ZCS2, (c) ZCS4, (d) ZCS6, (e) ZCS8, and (f) ZCS10 for band gap.

The calculated band gap of ZG, ZCG2, ZCG4, ZCG6, ZCG8 and ZCG10 are 3.53, 3.51, 3.49, 3.48, 3.46 and 3.45 eV, respectively, which also indicates the band gap of nanocomposites are decrease with the graphene (Figure S4).

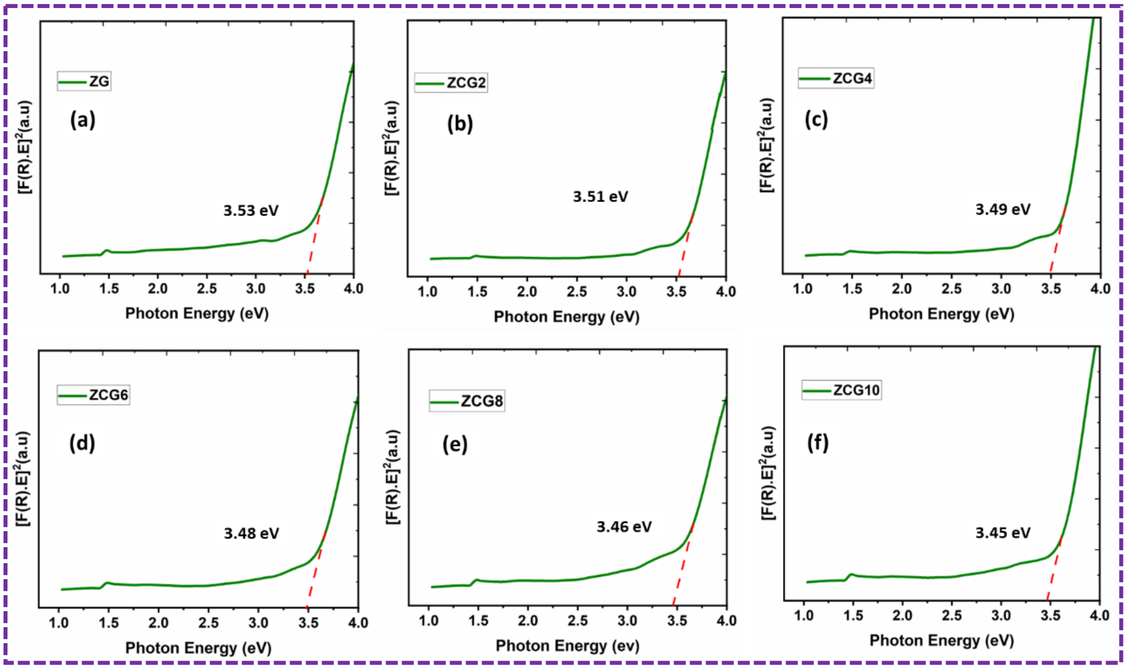

Figure S4. UV-vis DRS of (a) ZG. (b) ZCG2, (c) ZCG4, (d) ZCG6, (e) ZCG8, and (f) ZCG10 for band gap.

Fourier transform infrared spectroscopy (FTIR)

FT-IR analysis was performed for as-prepared samples **(Figure 5).** The surface of graphene sheets exhibited oxygen functional groups. The sample exhibits distinct bands at 3433.4, 2093.4, 1614.2, and 1095.8 cm^{-1}. The presence of absorbed water molecules was reason for the wide absorption bands observed at 3433.4 cm^{-1}, which can be indicated to bending and stretching vibrations of the O–H bonds. The presence of CO_2 molecules is attributed to the peaks observed at 2093 cm^{-1} [2]The band at 1614.2 cm^{-1} corresponds to presence of the functional group known as double bond C=C carboxylate stretching vibration. The vibration at 1095.8 cm^{-1} is indicated presence of C-O functional group indicates stretching of acetyl group. Generally, it can be stated that sample doping can lead to alterations in the peaks of the ZnS FT-IR spectrum, including the production of new bands and shifts in the location of some bands [3]. Moreover, it will be favourable for the stability of ZnS nanoparticles on the graphene sheet [4]. **Table 1** represents the peak positions, functional group and mode of vibrations of ZCG nanocomposite.

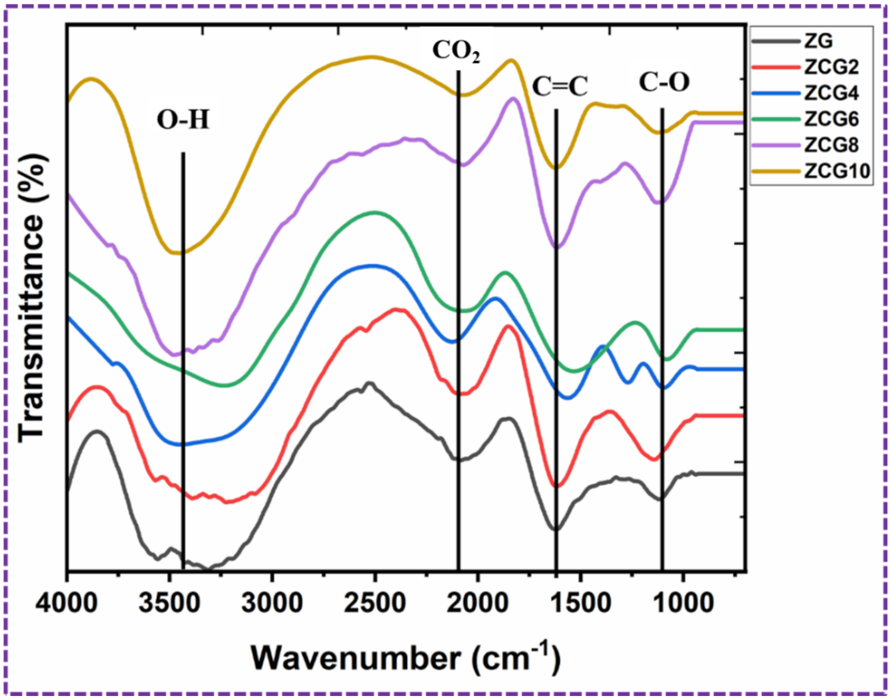

Figure S5. FTIR Spectrum of ZG, ZCG2, ZCG4, ZCG6, ZCG8, and ZCG10.

Table 1. FT-IR peak position, functional group and mode of vibration of ZCG nanocomposite.

Peak Position on FTIR spectra (cm^{-1})	Functional Group	Vibration Modes	Ref
3433.4	O-H	stretching and bending vibrations	[2]
2093	CO_2	CO_2 molecules	[2]
1614.2	C=C	carboxylate stretching vibration	[3]
1095.8	C-O	Stretching of acetyl group	[3]

Figure S6 shows that the cyclic performance of ZCG10 electrode after 10,000 cycles. Capacitive retention and coulombic efficiency o ZCG10 electrode are 36 % and 61 % after 10,000 cycles.

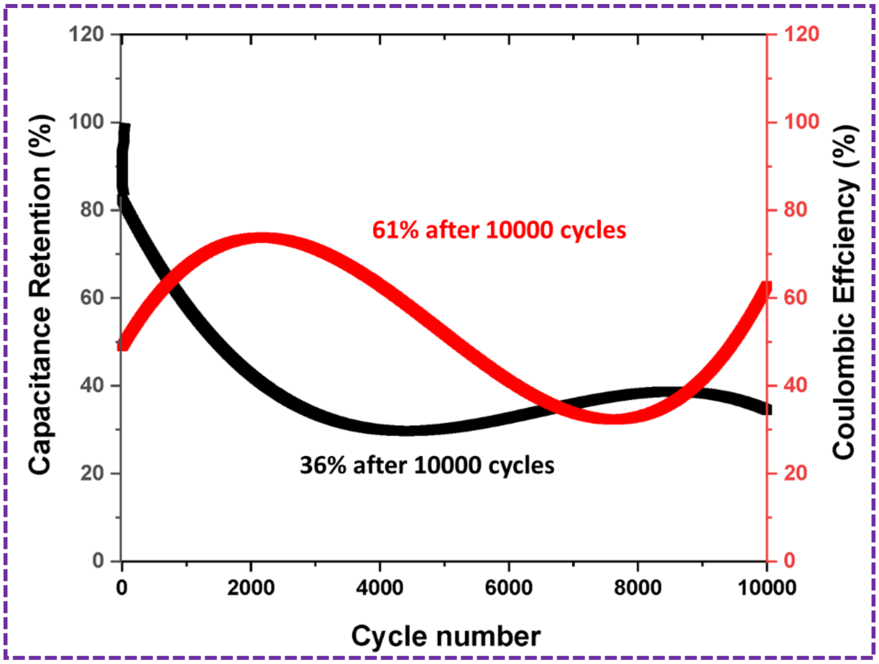

Figure S6. Cyclic performance of ZCG10 electrode after 10,000 cycles.

References

1. Kumari, P., et al., *Interrelation of micro-strain, energy band gap and PL intensity in Ce doped ZnS quantum structures.* Journal of Luminescence, 2022. **251**: p. 119258.
2. Jothibas, M., et al., *Structural and optical properties of zinc sulphide nanoparticles synthesized via solid state reaction method.* Journal of Materials Science: Materials in Electronics, 2017. **28**: p. 1889-1894.
3. Nikzad, M., M.R. Khanlary, and S. Rafiee, *Structural, optical and morphological properties of Cu-doped ZnS thin films synthesized by sol–gel method.* Applied Physics A, 2019. **125**: p. 1-9.
4. Sagadevan, S., et al., *One pot synthesis of hybrid ZnS–Graphene nanocomposite with enhanced photocatalytic activities using hydrothermal approach.* Journal of Materials Science: Materials in Electronics, 2018. **29**: p. 9099-9107.
